# Comparative transcriptome analyses of oleaginous *Botryococcus braunii* race A reveal significant differences in gene expression upon cobalt enrichment

**DOI:** 10.1186/s13068-018-1331-5

**Published:** 2018-12-18

**Authors:** Pengfei Cheng, Chengxu Zhou, Yan Wang, Zhihui Xu, Jilin Xu, Dongqing Zhou, Yinghui Zhang, Haizhen Wu, Xuezhi Zhang, Tianzhong Liu, Ming Tang, Qiyong Yang, Xiaojun Yan, Jianhua Fan

**Affiliations:** 1 College of Food and Pharmaceutical Sciences, Ningbo University, Ningbo, 315211 People’s Republic of China; 2 State Key Laboratory of Bioreactor Engineering, East China University of Science and Technology, 130 Meilong Road, Shanghai, 200237 People’s Republic of China; 3 Department of Applied Biology, East China University of Science and Technology, Shanghai, 200237 People’s Republic of China; 4 Institute of Hydrobiology, Chinese Academy of Sciences, Wuhan, 430072 People’s Republic of China; 5 Key Laboratory of Biofuels, Qingdao Institute of Bioenergy and Bioprocess Technology, Chinese Academy of Sciences, Qingdao, 266101 People’s Republic of China; 6 Poyang Lake Eco-economy Research Center, Jiujiang University, Jiujiang, 332000 People’s Republic of China; 7 Key Laboratory of Marine Biotechnology of Zhejiang Province, Ningbo University, 818 Fenghua Road, Ningbo, 315211 People’s Republic of China

**Keywords:** *Botryococcus braunii*, Cobalt treatment, Physiological response, Transcriptome, Regulation mechanism

## Abstract

**Background:**

*Botryococcus braunii* is known for its high hydrocarbon content, thus making it a strong candidate feedstock for biofuel production. Previous study has revealed that a high cobalt concentration can promote hydrocarbon synthesis and it has little effect on growth of *B. braunii* cells. However, mechanisms beyond the cobalt enrichment remain unknown. This study seeks to explore the physiological and transcriptional response and the metabolic pathways involved in cobalt-induced hydrocarbon synthesis in algae cells.

**Results:**

Growth curves were similar at either normal or high cobalt concentration (4.5 mg/L), suggesting the absence of obvious deleterious effects on growth introduced by cobalt. Photosynthesis indicators (decline in Fv/Fm ratio and chlorophyll content) and reactive oxygen species parameters revealed an increase in physiological stress in the high cobalt concentration. Moreover, cobalt enrichment treatment resulted in higher crude hydrocarbon content (51.3% on day 8) compared with the control (43.4% on day 8) throughout the experiment (with 18.2% improvement finally). Through the de novo assembly and functional annotation of the *B. braunii* race A SAG 807-1 transcriptome, we retrieved 196,276 non-redundant unigenes with an average length of 1086 bp. Of the assembled unigenes, 89,654 (45.7%), 42,209 (21.5%), and 32,318 (16.5%) were found to be associated with at least one KOG, GO, or KEGG ortholog function. In the early treatment (day 2), the most strongly upregulated genes were those involved in the fatty acid biosynthesis and metabolism and oxidative phosphorylation, whereas the most downregulated genes were those involved in carbohydrate metabolism and photosynthesis. Genes that produce terpenoid liquid hydrocarbons were also well identified and annotated, and 21 (or 29.2%) were differentially expressed along the cobalt treatment.

**Conclusions:**

*Botryococcus braunii* SAG 807-1 can tolerate high cobalt concentration and benefit from hydrocarbon accumulation. The time-course expression profiles for fatty acid biosynthesis, metabolism, and TAG assembly were obtained through different approaches but had equally satisfactory results with the redirection of free long-chain fatty acid and VLCFA away from TAG assembly and oxidation. These molecules served as precursors and backbone supply for the fatty acid-derived hydrocarbon accumulation. These findings provide a foundation for exploiting the regulation mechanisms in *B. braunii* race A for improved photosynthetic production of hydrocarbons.

**Electronic supplementary material:**

The online version of this article (10.1186/s13068-018-1331-5) contains supplementary material, which is available to authorized users.

## Background

Microalgae have become an excellent source of raw materials for biodiesel and value-added products due to its unique composition and structure [1, 2]. *Botryococcus braunii* is known for its high hydrocarbon production. Different from other algae, the hydrocarbons synthesized by *B. braunii* are more suitable in high-quality fuel applications, because they are rich in saturated fatty acids, monounsaturated fatty acids, and long-chain aliphatic hydrocarbons [3, 4]. Hydrocarbons synthesized by *B. braunii* are stored on the outer cell wall, making hydrocarbon oil extractions more economical and more convenient [3, 5].

*Botryococcus braunii* has three different races (A, B, L) based on the synthesized hydrocarbons. Race A produces fatty acid-derived C_23_–C_33_ alkadienes and alkatrienes and exhibits the largest range in hydrocarbon content among the three races [6]. However, *B. braunii*’s slow growth and long generation time, as well as our lack of a clear understanding of hydrocarbon anabolic pathways, restrict large-scale cultivation and application of *B. braunii* as a high-yield hydrocarbon feedstock [3]. Therefore, strengthening the research on the regulation of growth and production of hydrocarbons is important for the development and application of hydrocarbon-producing fuels.

Most of the methods used to improve hydrocarbon production in *B. braunii* are similar to other energy microalgae, which have high CO_2_ concentrations, high light intensity, or low limitation of nitrogen source. However, *B. braunii* grows slowly, and its final hydrocarbon yield is not ideal under the aforementioned conditions [7, 8]. *B. braunii* race A promotes the accumulation of oleic acid, and hydrocarbon content does not change much in the absence of nitrogen source conditions [9, 10]. Fang et al. [11] analyzed the transcriptome of *B. braunii* race A in nitrogen-lacking condition and found that the reason for *B. braunii* slow growth might be the energy flow to the lipid synthesis instead of cell growth. However, the specific reasons for slow growth and key metabolic pathways for hydrocarbon synthesis of *B. braunii* remain uncertain. Therefore, we need to seek new methods that can help us learn the key anabolic pathways of *B. braunii* race A, explain the mechanisms for slow growth in *B. braunii*, and provide the basis for improving hydrocarbon production in *B. braunii*.

In our previous studies, a strain hydrocarbon-producing *B. braunii* race A SAG 807-1 was cultured in normal medium supplemented with different concentrations of cobalt. The result revealed that a certain high concentration of cobalt can promote hydrocarbon synthesis in *B. braunii*, and it has small effect on the growth of *B. braunii* cells [12]. The results showed that the biomass productivity of *B. braunii* in a culture period of day 8 was similar, corresponding to the cobalt concentration of 0.09, 0.18, 0.45, 0.90 and 4.5 mg/L. However, the biomass was hindered when the concentration of cobalt increased. Cobalt is an important transition metal material. It is widely used in the production of lithium batteries, superalloys, insulating materials, and industrial catalysts [13, 14]. Under certain high concentrations of cobalt, *B. braunii* race A increases its hydrocarbon content and does not change its growth that much. This finding may imply that studying the high-producing hydrocarbon properties of *B. braunii* can be used to explore the feasibility ideas of its key synthesis pathways.

In general, under suitable conditions, microalgae mainly synthesize fatty acids in the form of glycolipids and phospholipids. The algae cells can change their lipid synthesis pathways, produce a large amount of neutral lipids, and maintain the normal metabolism under unfavorable growth conditions [15]. In addition, the key metabolic pathways for hydrocarbon synthesis in *B. braunii* race A are not clear. Therefore, based on the high hydrocarbon-producing characteristics of *B. braunii* race A under high cobalt concentration conditions, we can study the changes in key enzyme and genes and important metabolic pathways involved in *B. braunii* race A hydrocarbon synthesis.

However, the genome size of *B. braunii* race A is large. At present, most genomes of species are not fully sequenced, and we relatively lack the cognition of molecular biology of *B. braunii* [16–18]. Under unknown genomic information, transcriptome sequencing (mRNA-seq) can be used to discover the gene type and expression levels of cells in different conditions. Thus, we can obtain information on gene expression under the stress condition, infer the functions of the corresponding unknown gene, and reveal regulation gene action mechanisms. Therefore, the transcriptome analysis of *B. braunii* race A before and after cobalt stress provides techniques to understand the changes in key enzyme and genes in hydrocarbon synthesis in *B. braunii* race A, study the critical path of hydrocarbon production of *B. braunii* race A under high cobalt concentration, and may further expound the slow growth mechanism in *B. braunii*.

In summary, this paper is based on the growth characteristics of *B. braunii* and mechanisms of cobalt enrichment. We can study the physiological response characteristics and high hydrocarbon-producing characteristics of *B. braunii* race A. We can also elucidate the key metabolic pathways and metabolic regulations in *B. braunii* race A hydrocarbon synthesis. This study takes a strain hydrocarbon-producing *B. braunii* race A (*B. braunii* SAG 807-1) as the research subject and explores the photosynthetic characteristics of *B. braunii* under cobalt stress. RNA-seq was used to perform transcriptome sequencing analysis of *B. braunii* samples before and after cobalt stress and explore the metabolic pathways involved in the synthesis of hydrocarbons in algae cells induced by cobalt.

## Results

### Growth characteristics and chlorophyll content variation of *B. braunii* with the change of cobalt concentration

**Fig. 1:**
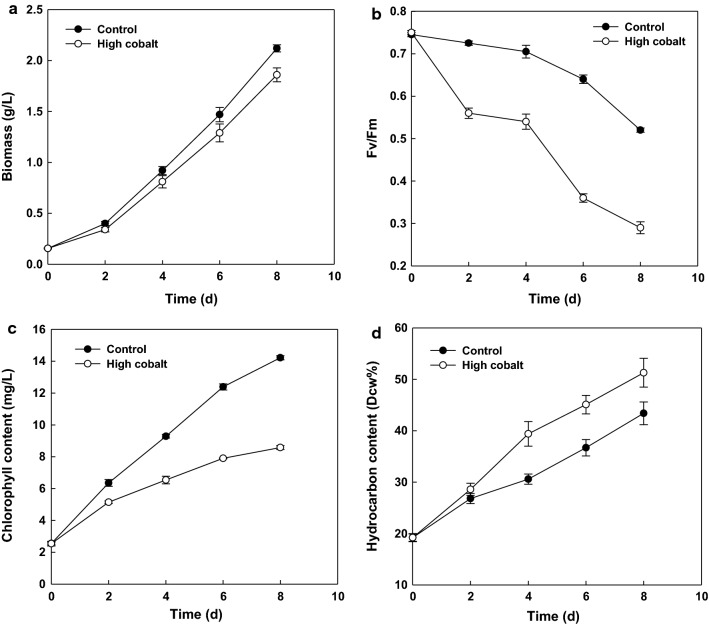
Comparison of growth and physiological response of *B. braunii* SAG 807-1 under cobalt enrichment treatment. **a** Dry cell weight; **b** variable-to-maximum fluorescence ratio (Fv/Fm); **c** chlorophyll content; **d** hydrocarbon content. Normal cobalt (control, 0.09 mg/L); high concentration cobalt (4.5 mg/L)

Autotrophic algal cells were collected and inoculated into modified Chu 13 medium for normal cobalt (control, 0.09 mg/L) and into high concentration cobalt (4.5 mg/L) with an initial biomass density of 0.16 g/L. The photoautotrophic growth rate of the algae was examined under 100 µmol/m^2^/s. As indicated in Fig. 1a, the growth of *Botryococcus* cells was not significantly different with the two treatments. However, at day 8, the biomass of the algae with high cobalt was 1.86 g/L, which was slightly lower than the control (2.12 g/L).

The chloroplast is the fundamental unit for most photosynthetic algae; hence the content of chlorophyll and the vitality of the photosynthetic machine are critical physiological indicators through which we can inspect algal cell adaptation when exposed to cobalt enrichment [5]. Figure 1c shows that the chlorophyll content of algal cells in normal Chu 13 medium cultivation fluctuated around 14.22 mg/L at day 8, whereas high cobalt concentration cultivations resulted in significant decrease in chlorophyll content (little difference was found in the two treatments at the beginning of the culture). The amount of *B. braunii* cells in high cobalt culture was 8.58 mg/L at 8 days. The degradation caused by cobalt enrichment was relatively less significant at the end of the culture.

The variable-to-maximum fluorescence ratio (Fv/Fm), which indicates about the quantum efficiency of photosystem II was also measured to evaluate changes in the photosynthetic efficiency of algal cell under the two treatments (Fig. 1b). In the normal Chu 13 medium cultivations (control), the Fv/Fm ratio increased on the first day then decreased gradually to 0.52 on day 8. The exposure of algal cells to cobalt enrichment led to a more dramatic decline in Fv/Fm ratio than in the control. The algae cells with high cobalt exhibited an early declining pattern with the terminal value of 0.29 at day 8. The decline in the Fv/Fm ratio value under cobalt enrichment indicates that the photosynthetic efficiency of algal cells was undermined. Nutrient stress was also demonstrated to lead to a decline in the Fv/Fm ratio [19].

### Hydrocarbon accumulation of *B. braunii* with the change in cobalt concentration

*Botryococcus braunii* is a colonial green microalga that can produce extracellular hydrocarbons at a high rate [4, 16]. However, hydrocarbon accumulation apparently does not require specific metabolic triggers like nitrogen starvation as seen in TAG-accumulating microalgae [9]. In this study, as shown in Fig. 1d, cobalt enrichment treatment resulted in higher content of crude hydrocarbon compared with the control throughout the experiment. Moreover, crude hydrocarbon content increased with the culture time. On day 8, the control reached a maximum crude hydrocarbon content of 43.4%, whereas the cobalt enrichment samples reached a maximum of 51.3%.

**Fig. 2:**
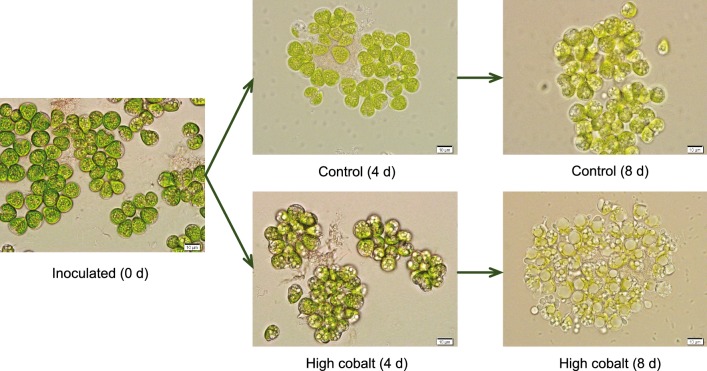
Light microscopic analysis of *B. braunii* SAG 807-1 under cobalt enrichment treatment. Normal cobalt (control, 0.09 mg/L); high concentration cobalt (4.5 mg/L)

The hydrocarbon composition produced by the strain suggested that it is a typical strain of the A race [6]. Electron microscopy also revealed that the algal cells, which were cultivated in the normal culture medium, were relatively fresh green (Fig. 2). More round lipid droplets (brackets) can also be seen attached to the cell surface under the cobalt enrichment culture. Round lipid droplets on the cell surface were considered to be lipids recently secreted to the cell surface. Lipid bodies in the cytoplasm were not prominent in interphase cells. These lipid bodies then increased in number, size, and inclusions, reaching maximum values just before the first lipid accumulation on the cell surface at the cell apex (Fig. 2). However, TAG conversion to biofuel crude usually involves transesterification of the constituent fatty acids with alcohols before refining the resulting biocrude to transportation fuels [20].

### Activities of antioxidant enzymes and antioxidant response

**Fig. 3:**
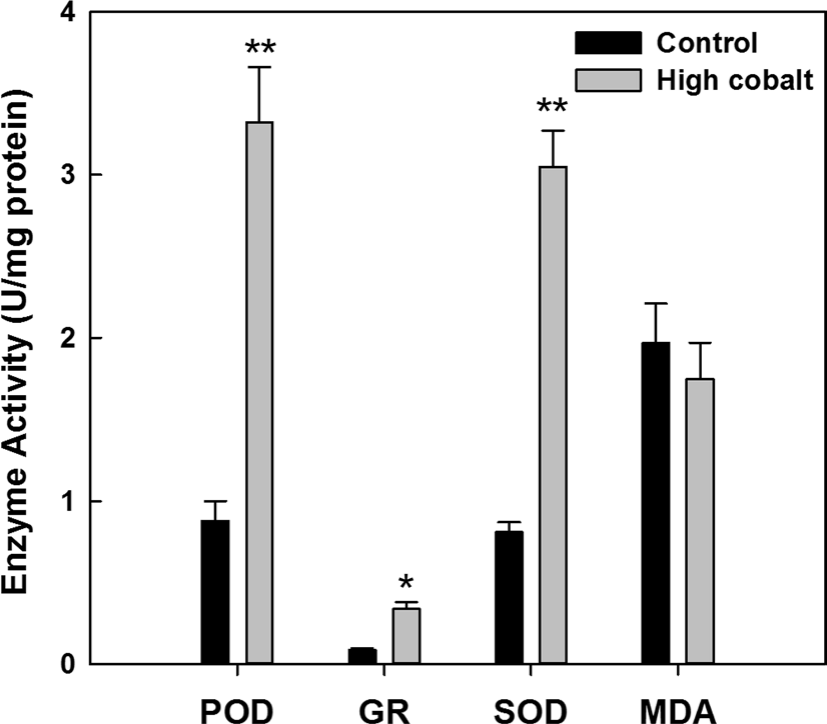
Activities of antioxidant enzymes like peroxidase (POD), glutathione reductase (GR), superoxide dismutase (SOD), and malonaldehyde (MDA) in *B. braunii* SAG 807-1 under cobalt enrichment treatment. Normal cobalt (control, 0.09 mg/L); high concentration cobalt (4.5 mg/L)

In the present study, we investigated physiological parameters to confirm the toxicological effects of the high cobalt stressor, including the activities of antioxidant enzymes like peroxidase (POD), glutathione reductase (GR), superoxide dismutase (SOD), and malonaldehyde (MDA) (Fig. 3). The activities of POD, GR, and SOD for algal cells, which were cultured in the cobalt enrichment medium were prominent, and the values were 3.32, 0.34, and 3.05 U/mg protein, respectively. Counterintuitively, the enzyme activity of POD, GR, and SOD under normal medium was even lower than that of high cobalt conditions at the late phase of cultivation. However, MDA content reflecting lipid peroxidation and no significant difference was found between the samples. We can figure out that the cell growth curve has an inverse trend with that of POD, GR, and SOD content. Researches indicated that the stress on green algae is mediated by antioxidant enzymes which SOD, GR, POD were mainly involved [19]. Therefore, our data indicated that cobalt enrichment may play vital roles in acclimation through regulation of the expression of specific genes. In general, nitrogen, phosphate, or iron deficiency in the culture alters enzymes in the cells or results in enhanced lipids biosynthesis. However, these factors were insignificant in the study of hydrocarbon synthesis and metabolism for the *B. braunii* [10, 21].

### De novo assembly and functional annotation of the *B. braunii* transcriptome

To investigate the transcriptomic change upon cobalt stress, the sequencing, assembly, and annotation of the *B. braunii* SAG 807-1 were carried out. A total of 659 million filtered reads were yielded through Illumina HiSeq 2000 platform, which represent the transcripts from total RNA isolated from four time points (days 0, 2, 4, 8) during the high cobalt and normal culture conditions (Additional file 1: Tables S1–S3). These time points were chosen because the difference of growth characteristics and enzyme activities have been shown to be significant in these time points. As a result, 196,276 non-redundant unigenes were obtained with an average length of 1086 bp, N50 of 1419 bp, and GC content of 51.76% (Table 1). These cleaned reads were deposited into the NCBI Sequence Read Archive (Accession: SRP161189).

**Table 1:** The de novo-assembled parameters of *B. braunii* SAG 807-1 transcriptome

Statistics	Counts	Total length (bp)	*N*25 (bp)	*N*50 (bp)	*N*75 (bp)	Average length (bp)	Longest (bp)	*N* %	GC %
Contigs	471,885	279,466,923	1893	784	395	592	17,019	0.60	51.50
Transcripts	208,740	218,861,772	2825	1327	677	1048	18,583	0.78	51.75
Unigenes	196,276	213,199,466	2964	1419	706	1086	18,869	0.76	51.76

Fourteen samples were collected from the bioreactors at 0, 2, 4, and 8 days at both cobalt enrichment and normal conditions

The whole genome sequence of *B. braunii* race A is not available, thus, the annotation of the non-redundant unigenes were performed based on the BlastX tool by sequence homology comparison. As a result, a total of 76,002 (38.7%) and 84,847 (43.2%) of the unigenes in SAG 807-1 were shown to have best-hits upon NCBI non-redundant protein database and UniProt, respectively (i.e. cut-off *E*-value ≤ 10^−5^) (Additional file 1: Table S4 and Additional file 2: Dataset S1). Among the annotation dataset, we found that the top five best-hits species were *Volvox carteri*, *Chlamydomonas reinhardtii*, *Ectocarpus siliculosus*, *Guillardia theta* CCMP2712, and *Chlorella variabilis*, suggesting that the transcriptome of SAG 807-1 is mostly related to green algae. The majority (top 20) of the best-hits species were listed in Additional file 1: Figure S2. Our result was supported by the annotation results. However, a slight difference was observed between another *B. brauni* race A strain 779, of which ~ 70% of best-hit ESTs were derived from green microalgae *Coccomyxa subellipsoidea* [11], implying that race A 779 and SAG 807-1 belong to different isolines of *B. brauni*.

The assembled unigenes were then subjected to functional analysis. Of the 196,276 ESTs, 89,654 (45.7%), 42,209 (21.5%) and 32,318 (16.5%) were found to be associated with at least one KOG (eukaryotic ortholog group), gene-ontology (GO), or KEGG ortholog function (Additional file 1: Figures S3–S5, Additional file 3: Dataset S2 and Additional file 4: Dataset S3), respectively. A total of 3682 GO functions in Biological Process, 648 functions in Cellular Component, and 2035 functions in Molecular Function were annotated in SAG 807-1 transcriptome (compete list were summarized in Additional file 3: Dataset S2). A complete list of unigene-associated KEGG metabolic pathways is collected in Additional file 4: Dataset S3.

### Global transcriptional changes of *B. braunii* in response to high cobalt

Individual unigenes levels were normalized to FPKM and averaged from two biological replicates for differential expression analysis. Compared with baseline, transcriptome data showed that 4472 unigenes were downregulated by twofold, whereas 3895 unigenes were upregulated twofold under high cobalt condition for 2 days. After 4-day stress, 7408 unigenes were downregulated, whereas 6541 unigenes were upregulated. After 8 days of treatment, 18,889 unigenes were downregulated, and 4197 unigenes were upregulated. The number of differentially expressed genes is presented in Additional file 1: Figure S6. Notably, under high cobalt conditions, a higher number of downregulated transcripts were observed compared with that of upregulated transcripts. At the peak of gene expression, the number of differentially expressed transcripts was clearly more than that of time points at days 2 and 4. Nonetheless, the overall pattern was that upregulation expression was highest at 4 days and lowest at 2 days, whereas the number of downregulated transcripts has gone up dramatically.

**Fig. 4:**
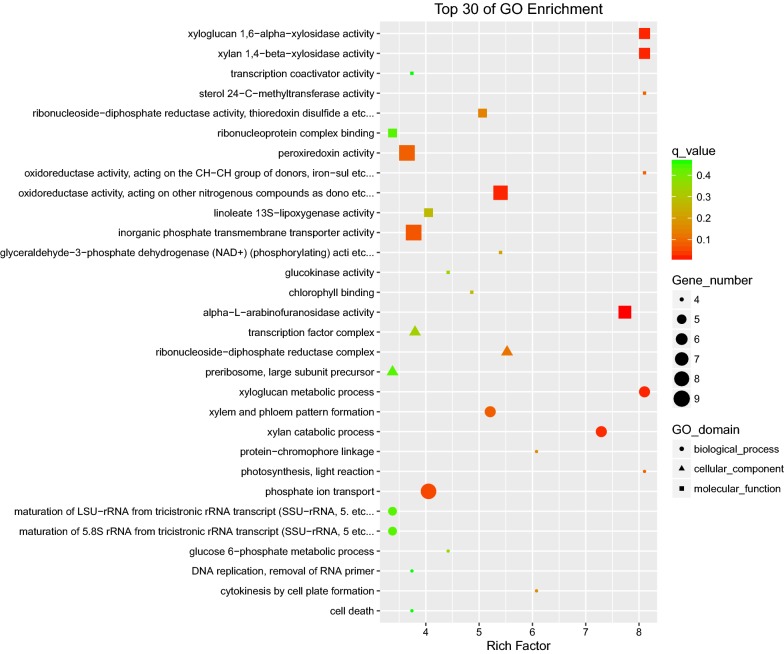
Distribution of top 30 GO categories enrichment upon high cobalt treatment (4 days). Enrichment were analyzed based on hypergeometric test and Bonferroni adjustment (corrected *P* value (FDR) ≤ 0.05)

**Fig. 5:**
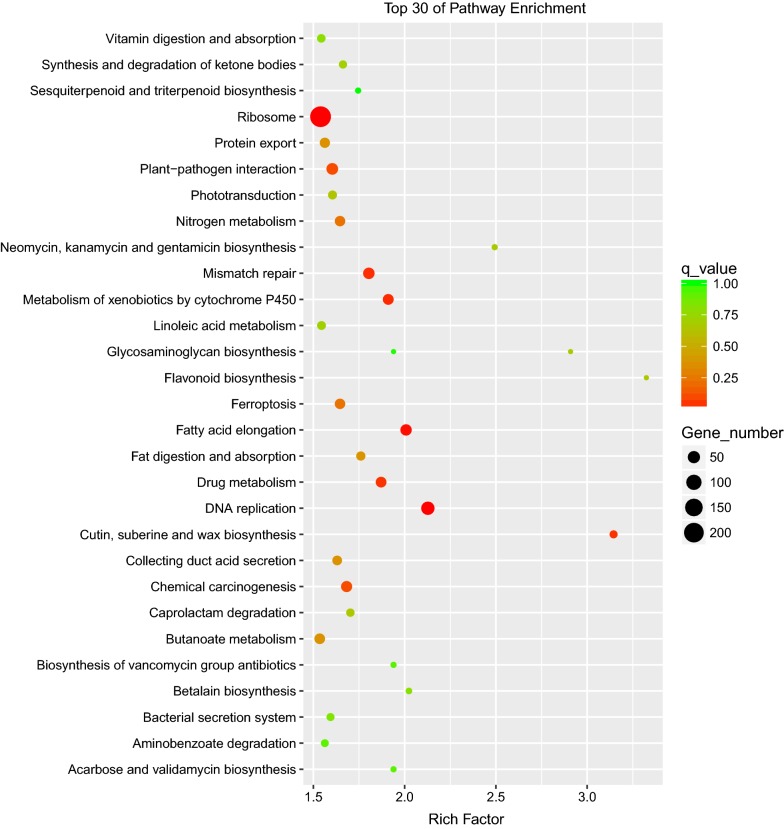
Distribution of top 30 KEGG metabolic pathways enrichment upon high cobalt treatment (4 days). Enrichment were analyzed based on hypergeometric test and Bonferroni adjustment (corrected *P* value (FDR) ≤ 0.05)

Transcriptomic dynamics tracked via mRNA-seq across three time points by comparison from high cobalt to normal conditions revealed genes and pathways that are most differentially expressed (Additional file 5: Dataset S4, Additional file 6: Dataset S5, Additional file 7: Dataset S6, Additional file 8: Dataset S7, Additional file 9: Dataset S8, Additional file 10: Dataset S9, Additional file 11: Dataset S10, Additional file 12: Dataset S11, Additional file 13: Dataset S12). For GO enrichment at 4 days, genes were those from the following: (1) biological processes: xyloglucan metabolic process, xylan catabolic process, xylem and phloem pattern formation, and phosphate ion transport; (2) cellular components: ribonucleoside-diphosphate reductase complex, transcription factor complex, and preribosome; and (3) molecular function: xyloglucan 1,6-alpha-xylosidase activity, xylan 1,4-beta-xylosidase activity, alpha-l-arabinofuranosidase activity, oxidoreductase activity, and peroxiredoxin activity (Fig. 4). For 2 days and 8 days, the top 30 of GO enrichment were displayed in Additional file 1: Figures S7, S8. For KEGG pathway enrichment at 4 days, genes that are involved in fatty acid elongation, sesquiterpenoid and triterpenoid biosynthesis, and linoleic acid metabolism were mostly differentially expressed (Fig. 5). For 2 days and 8 days, the top 30 of KEGG pathway enrichment were displayed in Additional file 1: Figures S9, S10. Notably, in the early stage of cobalt treatment (2 days), pathways like fatty acid metabolism, fatty acid elongation, fatty acid biosynthesis, biosynthesis of unsaturated fatty acids, and photosynthesis fluctuated remarkably. This finding suggests a vigorous gene regulatory response upon high cobalt concentration, which can partly explain the rapid and massive changes in intracellular long chain fatty acid and hydrocarbon storage forms within the cobalt treatment.

**Fig. 6:**
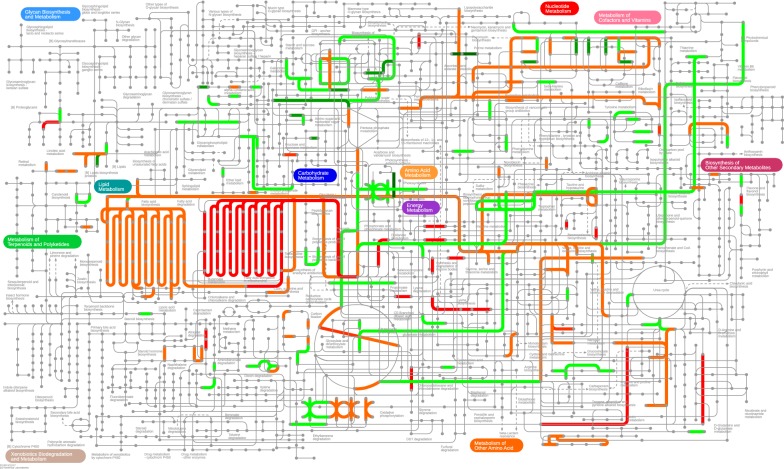
Overview of metabolic pathways and regulation during the high cobalt treatment (2 days) in *B. braunii* SAG 807-1. Pathways that are up- or down-regulated (compared with normal culture conditions) are labeled in red and green, respectively. Deeper red and green indicates greater fold changes of differential expression. These maps were visualized by iPath 3 interactive pathways explorer

To model the temporal process featuring the response of the intracellular gene expression profile upon high cobalt condition, iPath was applied to visualize and customize the various pathway maps using the KEGG Orthology (KO) database [22]. In the early growth process (2 days), the genes that were most strongly upregulated were those from the fatty acid biosynthesis and metabolism and oxidative phosphorylation, whereas the most downregulated ones were from carbohydrate metabolism and photosynthesis (Fig. 6). However, in the middle phase of growth (4 days), the genes that were most strongly upregulated were those from the oxidative phosphorylation and fatty acid degradation, whereas the most downregulated ones were from central carbon metabolism, photosynthesis, and amino acid metabolism (Additional file 1: Figure S11). At the stabilization phase (8 days), cobalt enrichment highlights the increased expression of genes involved in nucleotide metabolism and metabolism of cofactors and vitamins. By contrast, lipid metabolism, central carbon metabolism, pentose phosphate pathway, oxidative phosphorylation, and amino acid metabolism genes were dramatically transcriptionally downregulated (Additional file 1: Figure S12).

Time course distribution of up- and downregulated genes within the KEGG pathway collectively result in a marked redirection of metabolism under high cobalt treatment. High-level expression of these functions along the cobalt enrichment conditions in *B. braunii* cells suggests that the decline of Fv/Fm ratio (Fig. 1b) and significant increment of enzyme activity of POD, GR, and SOD (Fig. 3, consistent with the peroxiredoxin activity GO enrichment analysis in Fig. 4) were potentially owing to a major portion of its cellular fatty acid and hydrocarbon accumulation. Thus, our transcriptome data agree to the analytic conclusions showed by versatile changes of physiological parameter measurement (e.g. hydrocarbon accumulation, decline of photosynthesis efficiency, and antioxidant response).

### Differentially expressed genes related to fatty acid-derived hydrocarbon biosynthesis and TAG assembly

**Fig. 7:**
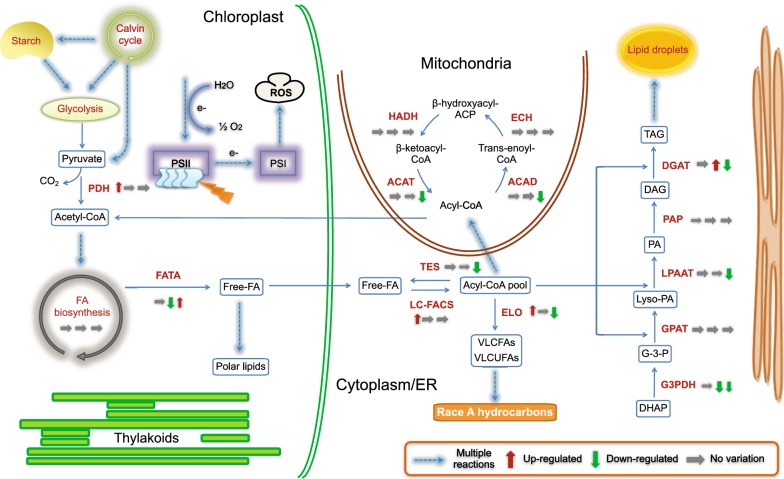
Fatty acid-derived hydrocarbon biosynthesis and lipid metabolism upon high cobalt treatment in *B. braunii* SAG 807-1. The pathways were constructed from transcriptomic evidence. Metabolic steps are represented by arrows. Dashed lines represent the presence of multiple metabolic steps. Products are shown in boxes. Genes encoding the enzymes in these pathways are labeled in red. Up- or down-regulation of gene expression under high cobalt conditions based on mRNA-seq data are indicated with red upward arrows and green downward arrows, respectively. The full names of the corresponding genes are given in Table 2. The left, middle and right arrows represent the 2 day, 4 day and 8 day cobalt treatments, respectively

Based on the subset of enzymes potentially involved in fatty acid-derived hydrocarbon biosynthesis and TAG assembly pathways, which were found in race A 779 [11], BOT-88-2 [23], and race B Showa [21], a total of 38 enzymes (association of with EC numbers) represented by 766 unigenes were retrieved in SAG 807-1 transcriptome (Table 2). The critical pathways related to fatty acid-derived hydrocarbon biosynthesis and lipid metabolisms were reconstructed from transcriptomic evidence (Fig. 7). Consistent with other two race A strains (779 and BOT-88-2), although the mRNA-seq data embraced putative genes related to unsaturated VLCFAs biosynthesis, the genes for the final conversion of VLCFAs to the hydrocarbon end products remain to be identified.

**Table 2:** List of annotated enzymes that potentially involved in fatty acid-derived hydrocarbon biosynthesis and TAG assembly

EC #	Enzyme description	Unigene number	LogFC (2 days)^a^	LogFC (4 days)	LogFC (8 days)
Fatty acid biosynthesis					
6.4.1.2	Acetyl-CoA carboxylase carboxyl transferase	17	0.16	− 0.51	0.12
6.3.4.14	Acetyl-CoA carboxylase/biotin carboxylase	27	− 0.17	− 0.61	0.04
2.3.1.39	[Acyl-carrier-protein] S-malonyltransferase	8	0.24	− 0.26	0.30
1.1.1.100	3-Oxoacyl-[acyl-carrier protein] reductase	52	0.01	− 0.21	− 0.25
2.3.1.179	3-Oxoacyl-[acyl-carrier-protein] synthase II	24	0.37	− 0.07	− 0.13
2.3.1.180	3-Oxoacyl-[acyl-carrier-protein] synthase III	6	0.83	0.31	− 0.58
1.14.19.2	Acyl-[acyl-carrier-protein] desaturase	6	0.40	0.21	− 0.03
1.14.19.1	Stearoyl-CoA desaturase (delta-9 desaturase)	23	0.95	0.39	− 0.69
3.1.2.14	Fatty acyl-ACP thioesterase A/oleoyl-ACP hydrolase	4	0.80	0.01	− 0.25
2.3.1.85	Fatty acid synthase, animal type	17	0.33	0.60	− 0.70
6.2.1.3	Long-chain acyl-CoA synthetase	95	0.14	− 0.22	− 0.10
6.2.1.3	Long-chain fatty acid-CoA ligase	10	*1.03*	0.98	− 0.74
3.1.2.21	Medium-chain acyl-[acyl-carrier-protein] hydrolase	1	− 0.15	*− 1.02*	*1.09*
Fatty acid desaturation and elongation					
1.14.19.6	Omega-6 fatty acid desaturase (delta-12 desaturase)	15	0.13	− 0.70	− 0.93
1.14.19.45	Omega-6 fatty acid desaturase (delta-12 desaturase)	16	0.81	0.09	0.09
2.3.1.199	3-Ketoacyl-CoA synthase	50	0.48	0.18	− 0.45
1.1.1.35	3-Hydroxyacyl-CoA dehydrogenase	5	*4.20*	*1.52*	*− 3.09*
1.1.1.211	Enoyl-CoA hydratase/long-chain 3-hydroxyacyl-CoA dehydrogenase	17	*2.53*	*1.44*	*− 1.23*
2.3.1.199	Fatty acid elongase	37	*1.11*	0.02	*− 1.32*
4.2.1.134	Very-long-chain (3R)-3-hydroxyacyl-CoA dehydratase	16	0.56	0.62	− 0.73
1.3.1.38	*Trans*-2-enoyl-CoA reductase	22	0.73	0.36	− 0.75
1.3.1.93	Very-long-chain enoyl-CoA reductase	9	0.75	0.37	− 0.55
Fatty acid oxidation					
4.1.1.9	Malonyl-CoA decarboxylase	7	− 0.04	0.06	− 0.44
2.3.1.16	Acetyl-CoA acyltransferase	33	0.69	0.82	− 0.67
3.1.2.2	Acyl-coenzyme A thioesterase	1	N.D.^b^	N.D.	− *3.05*
1.3.3.6	Acyl-CoA oxidase	53	0.22	0.05	− 0.10
1.3.99.12	Short/branched chain acyl-CoA dehydrogenase	7	0.26	0.16	− *2.4*
1.3.8.9	Very long chain acyl-CoA dehydrogenase	5	0.57	0.26	− *1.02*
1.3.8.7	Acyl-CoA dehydrogenase	25	0.08	− 0.14	− *2.43*
4.2.1.17	Enoyl-CoA hydratase	26	− 0.04	− 0.49	− 0.55
2.3.1.9	Acetyl-CoA C-acetyltransferase	28	0.46	0.40	− *1.28*
4.2.1.119	3-Hydroxyacyl-CoA dehydrogenase/enoyl-CoA hydratase 2	15	− 0.08	0.06	0.00
TAG synthesis					
2.7.1.30	Glycerol kinase	12	0.39	0.02	− 0.20
2.3.1.15	Glycerol-3-phosphate *O*-acyltransferase	17	0.04	− 0.30	− 0.27
2.3.1.51	Lysophospholipid acyltransferase	17	0.68	− 0.22	− *1.51*
3.1.3.4	Diacylglycerol diphosphate phosphatase/phosphatidate phosphatase	33	− 0.31	− 0.33	− 0.15
2.3.1.20	Diacylglycerol *O*-acyltransferase/wax-ester synthase	1	N.D.	*2.32*	*− 1.97*
2.3.1.158	Phospholipid:diacylglycerol acyltransferase	9	0.52	0.72	− 0.31

^a^ Summation of all FPKM values of unigenes that associated with the same enzyme were used for logFC calculation

^b^ N.D. means could not detect

^c^ More than twofold increase or decrease were marked as italic type

Accordingly, genes encoding enzymes associated with fatty acid and neutral lipid metabolic pathways were differentially expressed (Fig. 7). For comparison, the summation of all FPKM values of unigenes that associated with the same enzyme (based on EC number) were used for logFC calculation. The results revealed that the summative levels of the enzymes tested were moderately altered (Table 2). In the early growth process (2 days), four summative transcripts encoding long-chain fatty acid-CoA ligase (EC 6.2.1.3), 3-hydroxyacyl-CoA dehydrogenase (EC1.1.1.35), enoyl-CoA hydratase/long-chain 3-hydroxyacyl-CoA dehydrogenase (EC1.1.1.211), and fatty acid elongase (EC 2.3.1.199), which define the beginning committed steps of fatty acid biosynthesis (desaturation and elongation), were upregulated along the 4-day cobalt enrichment cultivation (i.e. fold change > 2) until the stabilization phase (8 days). Transcripts responsible for TAG assembly [e.g. diacylglycerol *O*-acyltransferase (EC 2.3.1.20)] showed remarkable increase in the middle phase of growth (4 days). Notably, five enzymes involved in fatty acid oxidation, including acyl-coenzyme A thioesterase (EC 3.1.2.2), short/branched chain acyl-CoA dehydrogenase (EC 1.3.99.12), very long-chain acyl-CoA dehydrogenase (EC 1.3.8.9), acyl-CoA dehydrogenase (EC 1.3.8.7), and acetyl-CoA C-acetyltransferase (EC 2.3.1.9) were all downregulated by the end of high cobalt treatment (8 days). Besides, three enzymes related to TAG synthesis, glyceraldehyde-3-phosphate dehydrogenase (EC 1.2.1.12), lysophospholipid acyltransferase (EC 2.3.1.51), and diacylglycerol *O*-acyltransferase (EC 2.3.1.20) also showed significant downregulation in the stabilization phase (8 days). However, the pyruvate dehydrogenase (E1), which serves as a critical gene of energy and carbon molecule supply for fatty acid biosynthesis, showed increased expression in the early cobalt treatment (2 days). The expression profile of the fatty acid biosynthesis, metabolism and TAG assembly were obtained through different approaches but had equally satisfactory results with the redirection of free long-chain fatty acid and VLCFA away from TAG assembly and oxidation, but served as precursors and backbone supply for the fatty acid-derived hydrocarbon accumulation.

### Differentially expressed genes involved in terpenoid liquid hydrocarbon biosynthesis pathways

Genes and metabolic pathways that produce terpenoid liquid hydrocarbons (e.g. botryococcene or squalene) in *B. braunii* race B Showa were well-identified and comprehensively annotated [21, 24]. To examine whether the transcription of enzymes involved in biosynthesis of botryococcene or squalene biosynthesis pathways were changed in high cobalt conditions and when unigenes between *B. braunii* SAG 807-1 and Showa were compared based on the association with EC numbers. Overall, 72 enzymes associated with 762 unigenes were found to be related to curated botryococcene and squalene biosynthesis pathways in Showa [21]. In line with the summation of unigene FPKM values with the same enzyme, 21 (or 29.2%) were differentially expressed along the cobalt treatment (i.e. fold change > 2, Table 3).

**Table 3:** List of annotated enzymes that potentially involved in botryococcene or squalene biosynthesis

EC #	Enzyme description	Unigene number	LogFC (2 days)^a^	LogFC (4 days)	LogFC (8 days)
IPP and DMAPP biosynthesis					
2.3.1.9	Acetyl-CoA C-acetyltransferase	28	0.46	0.40	*− 1.28*
2.3.3.10	Hydroxymethylglutaryl-CoA synthase	12	0.48	0.50	− 0.98
1.1.1.34	Hydroxymethylglutaryl-CoA reductase (NADPH)	15	0.55	0.31	*− 1.15*
2.7.1.36	Mevalonate kinase	5	*3.44*	-0.21	*− 3.20*
4.1.1.33	Diphosphomevalonate decarboxylase	7	*2.16*	0.23	*− 2.48*
2.7.2.3	Phosphoglycerate kinase	27	0.03	− 0.01	− 0.33
1.2.1.12	Glyceraldehyde 3-phosphate dehydrogenase	40	0.04	− 0.29	− 0.46
1.2.1.9	Glyceraldehyde-3-phosphate dehydrogenase (NADP+)	2	0.35	0.94	− 0.99
5.4.2.12	2,3-Bisphosphoglycerate-independent phosphoglycerate mutase	9	− 0.32	− 0.34	− 0.51
5.4.2.11	2,3-Bisphosphoglycerate-dependent phosphoglycerate mutase	10	*1.41*	0.28	− *1.90*
4.2.1.11	Enolase	32	− 0.18	− 0.49	− 0.80
2.7.1.40	Pyruvate kinase	55	0.47	− 0.14	− 0.96
2.2.1.7	1-Deoxy-d-xylulose-5-phosphate synthase	6	0.00	− 0.25	− 0.35
1.1.1.267	1-Deoxy-d-xylulose-5-phosphate reductoisomerase	5	0.25	0.21	− 0.39
2.7.7.60	2-C-methyl-d-erythritol 4-phosphate cytidylyltransferase	3	− 0.59	0.09	− 0.62
2.7.1.148	4-Diphosphocytidyl-2-C-methyl-d-erythritol kinase	4	− 0.05	− 0.22	*− 1.35*
4.6.1.12	2-C-methyl-d-erythritol 2,4-cyclodiphosphate synthase	5	− 0.37	0.41	− 0.49
1.17.1.2	4-Hydroxy-3-methylbut-2-enyl diphosphate reductase	5	− 0.07	− 0.41	− 0.30
1.17.7.3	(E)-4-hydroxy-3-methylbut-2-enyl-diphosphate synthase	9	0.03	− 0.07	− 0.39
5.3.3.2	Isopentenyl-diphosphate delta-isomerase	7	0.48	0.01	− *1.07*
5.1.3.1	Ribulose-phosphate 3-epimerase	17	− 0.08	0.02	− 0.53
2.2.1.1	Transketolase	27	0.42	− 0.05	− 0.43
4.1.2.22	Xylulose-5-phosphate/fructose-6-phosphate phosphoketolase	3	0.52	N.D.^b^	N.D.
Polyprenyl diphosphate synthases					
2.5.1.1	Geranyl diphosphate synthase	1	0.68	− 0.38	− 0.09
2.5.1.10	Farnesyl diphosphate synthase	14	0.43	− 0.35	− *1.08*
2.5.1.29	Geranylgeranyl diphosphate synthase, type III	10	*1.44*	0.75	− *1.64*
2.5.1.29	Geranylgeranyl diphosphate synthase, type II	5	0.37	0.09	− 0.75
2.5.1.91	Decaprenyl-diphosphate synthase subunit 1	7	0.68	0.80	− *1.09*
Triterpenoid hydrocarbon biosynthesis					
2.5.1.21	Squalene synthase/farnesyl-diphosphate farnesyltransferase	12	− 0.19	− 0.48	− 0.68
2.1.1.41	Sterol 24-C-methyltransferase	32	0.28	− 0.45	− *1.26*
2.1.1.143	24-Methylenesterol C-methyltransferase	3	0.44	0.00	0.50
1.14.14.17	Squalene monooxygenase	8	0.47	0.05	− 0.22
Triterpenoid sterol biosynthesis					
5.4.99.8	Cycloartenol synthase	4	− 0.10	− 0.20	0.55
1.14.13.72	Methylsterol monooxygenase	17	0.52	0.68	− *1.37*
5.5.1.9	Cycloeucalenol cycloisomerase	7	0.85	0.20	− *1.06*
1.14.13.70	Sterol 14-demethylase	14	0.33	− 0.03	− 0.45
1.3.1.70	Delta14-sterol reductase	10	− 0.34	− 0.25	− 0.55
5.3.3.5	Cholestenol Delta-isomerase	5	0.51	0.05	0.13
1.3.1.21	Cholesterol 7-dehydrogenase	13	*2.10*	0.75	0.41
1.3.1.21	7-Dehydrocholesterol reductase	9	*1.37*	*1.45*	− *1.67*
1.1.1.170	Sterol-4alpha-carboxylate 3-dehydrogenase (decarboxylating)	12	− 0.76	− 0.54	− 0.24
1.14.13.159	Vitamin D 25-hydroxylase	1	− 0.86	− *4.87*	N.D.
3.1.1.13	Bile salt-stimulated lipase	2	− *1.03*	− *4.22*	N.D.
3.1.1.13	Lysosomal acid lipase/cholesteryl ester hydrolase	37	0.88	0.31	− 0.98
Tetraterpenoid biosynthesis					
2.5.1.32	Phytoene synthase	5	0.06	0.15	− *1.18*
1.3.5.5	15-*cis*-phytoene desaturase	6	0.42	0.31	− 0.18
1.3.5.6	Zeta-carotene desaturase	5	0.42	0.59	− 0.03
5.2.1.13	Prolycopene isomerase	4	0.30	0.08	− 0.19
5.5.1.18	Lycopene epsilon-cyclase	3	− 0.22	0.15	− 0.67
5.5.1.19	Lycopene beta-cyclase	5	0.34	0.24	0.10
1.14.13.129	Beta-carotene 3-hydroxylase	2	− 0.09	− 0.45	− 0.45
1.14.99.45	Carotene epsilon-monooxygenase	4	0.64	0.29	0.14
1.14.13.90	Zeaxanthin epoxidase	10	0.00	− 0.36	− 0.24
1.23.5.1	Violaxanthin de-epoxidase	4	− 0.17	0.00	− 0.59
Meroterpenoid quinone biosynthesis					
1.3.1.83	Geranylgeranyl reductase	7	0.30	0.37	− 0.62
2.5.1.62	Chlorophyll synthase	5	0.15	0.52	− 0.34
2.5.1.116	Homogentisate phytyltransferase/homogentisate geranylgeranyltransferase	5	0.86	0.53	− 0.24
5.5.1.24	Tocopherol cyclase	3	0.20	− 0.96	0.34
2.1.1.95	Tocopherol *O*-methyltransferase	9	− 0.03	− 0.67	0.14
2.5.1.117	Homogentisate solanesyltransferase	3	0.52	0.12	− 0.83
2.5.1.74	1,4-Dihydroxy-2-naphthoate octaprenyltransferase	3	0.39	0.64	− *1.29*
2.5.1.39	4-Hydroxybenzoate polyprenyltransferase	4	0.40	0.24	0.08
2.1.1.64	Polyprenyldihydroxybenzoate methyltransferase	7	− 0.73	*1.00*	− 0.40
1.14.13.-	Ubiquinone biosynthesis monooxygenase Coq7	2	− 0.17	− *3.14*	− *3.75*
1.14.13.-	Ubiquinone biosynthesis monooxygenase Coq6	6	0.97	0.07	− 0.05
2.1.1.201	2-Methoxy-6-polyprenyl-1,4-benzoquinol methylase Coq5	14	− 0.22	0.00	− 0.14
Biosynthesis of gibberellic acid diterpenes					
1.14.11.12	Gibberellin 20-oxidase	1	− *1.78*	− 0.37	− *1.18*
*S*-adenosylmethionine regeneration					
3.3.1.1	Adenosylhomocysteinase	27	0.14	− 0.32	0.19
2.1.1.10	Homocysteine *S*-methyltransferase	4	− 0.08	− 0.44	− 0.52
2.1.1.13	5-Methyltetrahydrofolate–homocysteine methyltransferase	24	0.23	0.07	− 0.71
2.1.1.14	5-Methyltetrahydropteroyltriglutamate–homocysteine methyltransferase	8	− 0.86	0.87	− 0.27
2.5.1.6	*S*-Adenosylmethionine synthetase	26	0.28	0.07	− 0.24

^a^ Summation of all FPKM values of unigenes that associated with the same enzyme were used for logFC calculation

^b^ N.D. means could not detect

^c^ More than twofold increase or decrease were marked as italic type

Unigenes encoding seven enzymes related to IPP and DMAPP biosynthesis, namely, acetyl-CoA C-acetyltransferase (EC 2.3.1.9), hydroxymethylglutaryl-CoA reductase (NADPH) (EC 1.1.1.34), mevalonate kinase (EC 2.7.1.36), diphosphomevalonate decarboxylase (EC 4.1.1.33), 2,3-bisphosphoglycerate-dependent phosphoglycerate mutase (EC 5.4.2.11), 4 diphosphocytidyl-2-C-methyl-d-erythritol kinase (EC 2.7.1.148), and isopentenyl-diphosphate delta-isomerase (EC 5.3.3.2) were altered transcriptionally either in the early phase (2 days) or in the stabilization phase (8 days). In polyprenyl diphosphate synthases pathways, three enzymes including farnesyl diphosphate synthase (EC 2.5.1.10), geranylgeranyl diphosphate synthase, type III (EC 2.5.1.29), and decaprenyl-diphosphate synthase subunit 1 (EC 2.5.1.91) showed fold decrease in mRNA abundance in stabilization phase (8 days). As with triterpenoid hydrocarbon biosynthesis, only sterol 24-C-methyltransferase (EC 2.1.1.41) was downregulated by the end of cultivation. However, for triterpenoid sterol biosynthesis, the expression of six enzymes like methylsterol monooxygenase (EC 1.14.13.72), cycloeucalenol cycloisomerase (EC 5.5.1.9), cholesterol 7-dehydrogenase (EC 1.3.1.21), 7-dehydrocholesterol reductase (EC 1.3.1.21), vitamin D 25-hydroxylase (EC 1.14.13.159), and bile salt-stimulated lipase (EC 3.1.1.13) were remarkably changed. The transcription level of enzymes involved in tetraterpenoid biosynthesis were not dramatically altered under high cobalt treatment, except for phytoene synthase (EC 2.5.1.32) (> twofold decrease). Three enzymes in the meroterpenoid quinone biosynthesis pathways were present, including 1,4-dihydroxy-2-naphthoate octaprenyltransferase (EC 2.5.1.74), polyprenyldihydroxybenzoate methyltransferase (EC 2.1.1.64), and ubiquinone biosynthesis monooxygenase Coq7 (EC 1.14.13.-), which were transcriptionally changed in a later period of culture. One gene related to biosynthesis of gibberellic acid diterpenes, gibberellin 20-oxidase (EC 1.14.11.12), showed a synchronized decrease along the cobalt enrichment condition. No variation was observed in genes related to the *S*-adenosylmethionine regeneration pathway. Overall, these differentially expressed unigenes mostly exhibited similar expression patterns, that is, transcriptionally upregulated at the early phase of cobalt enrichment, then recovered following 2 days of cultivation but downregulated quickly after a long time of treatment (8 days).

## Discussion

The high oil content of *B. braunii* makes it extremely economically valuable, and it can synthesize a variety of hydrocarbons. Hence, extracting the hydrocarbon is more convenient than other lipids secreted by other algae. More encouragingly, the cosmopolitan green colonial microalga *B. braunii* stores photosynthetic carbon in the form of liquid hydrocarbons, which need no chemical conversion to provide biofuel crude [25]. *B. braunii* belongs to three races defined by their synthesized hydrocarbon products. Strains of races A, B, and L are present. The hydrocarbons synthesized for each race are specific to this race. *B. braunii* race A strains mainly accumulate C_23_–C_33_ odd carbon diolefins or triolefins derived from very long chain fatty acids [6]. B race strains of *B. braunii* mainly accumulate triterpenoids (C_30_–C_37_ botryococcenes) [6] and methylated squalenes [26]. L race strains of *B. braunii* mainly accumulate the tetraterpene lycopadiene [6, 16]. Recently, studies have discovered new S race strains of *B. braunii*. Race S strains synthesize C_18_ alkylene oxides and C_20_ saturated alkanes [27]. Compared with hydrocarbons synthesized by *B. braunii* race A, their carbon chains are much shorter, and the number of carbon atoms is even. In our previous studies, Cheng et al. [12] reported that *B. braunii* SAG 807-1 can tolerate high cobalt concentration under biofilm attached culture. In this research, the algae with suspension culture could also adapt with the high concentration of cobalt. A certain low concentration of cobalt in medium could stimulate algae growth when relatively higher concentrations are toxic. The role of cobalt in photosynthesis is controversial. Its toxic effect takes place by the inhibition of PSII activity. It inhibits either the reaction center or component of PSII acceptor by modifying secondary quinone electron acceptor Q_B_ site [28].

In green algae and high plants, glycerolipids biosynthesis is presumably carried out by two distinct pathways, namely, the prokaryotic pathway (located in the chloroplast) and the eukaryotic pathway (which occurs in the endoplasmic reticulum) [29]. As expected, besides many unigenes associated with the prokaryotic pathway were found in SAG 807-1 transcriptome, and 17 unigenes named fatty acid synthase (animal type, EC 2.3.1.85) were also identified, which implies that fatty acid elongation might take place not only in the chloroplasts but also in the cytosol. A previous research indicated that an alternate pathway for TAG synthesis in yeast, plants, and green algae is present, and it involves phospholipid: diacylglycerol acyltransferase (PDAT) to generate TAG using phospholipids as donors. Interestingly, nine PDAT unigenes were retrieved in the SAG 807-1 transcriptome.

At present, the study of gene function in *B. braunii* is not perfect. Only the genome sequence of the Showa strain of *B. braunii* race B has been initially completed [24]. The transcriptome analysis of race A strains [11, 23], race B strains [21], and race L strains have been reported [16]. The average GC content estimated using all non-redundant sequences was 49.5% for *B. braunii* race A BOT-88-2 [23] and 50.8% for *B. braunii* race B Showa [24]. In this study, our results showed race A SAG 807-1 has an average GC content of 51.7% for unigenes (Table 1). Overall, *B. braunii* has a lower GC proportion than in other green algae (such as *Chlamydomonas* and *Chlorella*, which are great that 65% [30, 31]). Known transcriptomes and their annotation information have good reference information, which can help with subsequent detailed annotation of the Showa genome. Studies on key enzymes and genes involved in hydrocarbon synthesis are present but limited. The degenerate PCR primers were designed by Okada et al. [32] through the conserved amino acid sequence region of squalene synthase. The cDNA was synthesized by reverse transcription PCR and then was used as probes to obtain the cloned fragment of squalene synthase gene in B race strains of *B. braunii*.

The biosynthetic precursors of diolefins and triolefins in A race strains of *B. braunii* are long-chain fatty acids or ultra-long-chain fatty acids. At first, these precursors catalyzed by long-chain fatty acid coenzyme A ligase to form long-chain fatty coenzyme A. The long-chain fatty coenzyme A is further catalyzed by fatty acyl-CoA reductase to form long-chain fatty aldehydes [23]. Finally, the aldehyde decarboxylase catalyzes fatty aldehyde to form olefins having a terminal double bond or a medium chain double bond. However, some studies have isolated a decarboxylase containing cobalt porphyrin from *B. braunii* race A. The decarboxylase can catalyze fatty aldehydes to form alkanes [33].

Baba et al. [23] firstly analyzed the transcriptomes of *B. braunii* race A BOT-88-2 in 2012. They sequenced the constructed cDNA library with the Roche 454 pyrosequencing instrument. They discovered several genes involved in the hydrocarbon biosynthetic pathway through BlastX annotation. The non-redundant transcriptomes of *B. braunii* BOT-88-2 has 29,038 ESTs, and its fatty acid synthesis pathway is very active in fatty acid anabolism. Its fatty acid chain extends similar to bacteria and plants and is achieved by a series of single-function enzymes, namely, the type II system. In addition to fatty acid metabolism pathway, its glycerol phospholipid metabolic pathway is also very active, and the high oil content of *B. braunii* race A may be related to it. However, they have not found a key gene for the biotransformation of ultra-long-chain fatty acids to form the final hydrocarbon in *B. braunii* BOT-88-2. We speculate that the functional gene in *B. braunii* race A may be unique. Thus, finding a homologous gene to identify it is impossible.

Fang et al. [11] subsequently found a fast-growing *B. braunii* race A 779 and studied the regulation of nitrogen starvation on its transcriptomes in 2015. They took two samples before and after nitrogen starvation treatment and then sequenced them with Illumina Hiseq 2000 sequencer. Finally, they obtained 61,220 non-redundant transcriptomes. After BlastX comparison, they found 12,292 ESTs with homologous gene protein matching. The study found 20 enzymes those biosynthesize long-chain fatty acids in *B. braunii* 779. After comparing the *B. braunii* 779 transcriptomes with the *B. braunii* BOT-88-2 transcriptomes, they found that many unannotated homologous sequences are present in the two strains. This finding indicated that many unknown genes are waiting for exploring in *B. braunii* race A. Based on the de novo splicing transcriptomes, this study revealed the functional enrichment analysis of *B. braunii* 779 intracellular before and after nitrogen starvation treatment, the transcriptional regulation direction after nitrogen starvation treatment, and the functional comparison between transcriptomes.

Functional enrichment analysis of *B. braunii* 779 genomes revealed that ESTs involved in energy metabolism were abundantly expressed. The authors speculated that the slow growth of *B. braunii* may be its photosynthetic energy being used to synthesize oils and hydrocarbons [11]. After nitrogen starvation treatment, they found that the EST expression values involved in photosynthesis and ribosomal proteins are downregulated. Nitrogen starvation significantly increased the accumulation of oils and hydrocarbons in *B. braunii* 779. However, the long-chain fatty acid metabolism pathways of precursors for biosynthesis of diolefins and triolefins in *B. braunii* 779 were not significantly upregulated or downregulated.

Unlike race A strains, the botryococcenes and tetraterpenoids produced by the races B and L were produced by the terpenoid biosynthetic pathway [6, 21]. After analyzing two transcriptomes of *B. braunii* BOT-70 and BOT-22, Ioki obtained de-redundant transcriptomes containing 1868 and 27,427 ESTs, respectively [34, 35]. Combined with two transcriptome analyses, we found that the synthetic pathway of DMAPP and IPP was an MVI synthesis pathway independent of mevalonate, and no enzyme was related to the mevalonate synthesis pathway in the transcriptome. They did not find major botryococcene synthase enzymes involved in the synthesis of race B strains hydrocarbon end products in the *B. braunii* BOT-70 and BOT-22 transcriptomes.

Niehaus et al. [36] suggested that the catalytic synthesis mechanism of botryococcene hydrocarbon synthase should be similar to that of squalene synthase. Thus, they should have similar amino acid sequence domains. Compared with gene clone and transcriptome data analysis, they found three squalene synthase sequences, namely, SSL-1, SSL-2, and SSL-3 and described the catalytic mechanisms of these three enzymes in *B. braunii* race B. Molnar et al. [21] subsequently analyzed the transcriptomes of *B. braunii* races B Showa. They found that the measured quantity and quality were higher and better than other transcriptome data of *B. braunii* race B. The final number of spliced ESTs was 46,422. Subsequently, they reconstructed the *B. braunii* Showa biological metabolic pathway based on the annotations of the *B. braunii* Showa transcriptomes and the existing KEGG data. Finally, they gave the terpene precursor synthesis pathway, the steroid carbon skeleton synthesis pathway, the triterpenoid synthesis pathway, other anthraquinone synthesis pathways, triacylglycerol synthesis pathways, and starch synthesis pathways [21].

Recently, Thapa et al. [16] discovered two squalene synthase sequences in L race strains of *B. braunii* with transcriptome sequencing. One is LSS, which is the squalene synthase sequence in *B. braunii* race L. The other is LOS, which catalyzes the biosynthesis of lycopaoctaene synthase in *B. braunii* race L. The late discovery of S race strains of *B. braunii* led to no recent reports on its transcriptomes.

Under different concentrations of cobalt, *B. braunii* can establish a series of adaptation or tolerance mechanisms to maintain chlorophyll a good physiological functions. Photosynthetic response is one of the important tolerance mechanisms [37]. However, excessively high concentration of cobalt (45 mg/L) will hinder the growth of algae cells. One of the reasons is that some complex proteins on the chloroplasts of algal cells can enhance the light-absorbing ability of the photosynthetic system and promote cell growth during the photosynthetic process. However, excessively high concentrations of metal can damage complex proteins on the chloroplast. It may be due to lack of constructional matters for maintaining the photosynthetic machinery [38]. Thus, based on studies of the photosynthetic response characteristics of algal cells on the stress of cobalt, understanding the limiting factors of slow growth of *B. braunii* is helpful.

High concentrations of metals in the water can hinder the growth of algae cells, change the content of chlorophyll A, break the electron transport chain, and change the parameters, such as chlorophyll fluorescence yield and electron transfer chain rates [39, 40]. Previous study suggested that the PSII of the thylakoid membrane of algal cells is one of the most important membrane protein complexes of the chloroplasts of algal cells and that it is very sensitive to metal contamination [41]. If the thylakoid membrane is damaged, the photosynthetic efficiency of algae cells will be reduced. PSII is mainly composed of core compounds (core antennas CP43, CP47 and reaction centers D1, D2, and Cytb_559_), light-harvesting antenna, and peripheral proteins. *B. braunii* can better grow under a certain concentration of cobalt. However, excessively high concentrations of cobalt hinder the growth of algal cells. Does this mean that the corresponding components of thylakoid on the algal cells’ membrane respond to cobalt? Therefore, further studying the role of cobalt in the photosynthetic electron transport chain of the algae cells and the effect of high concentrations of cobalt on the polypeptide components of the thylakoid membranes of algal cells, elucidate the photosynthetic characteristics of tolerance to high concentrations of cobalt in *B. braunii*, and further understand the limiting factors of the slow growth of *B. braunii* is necessary.

## Conclusions

*Botryococcus braunii* is a unicellular green alga with a simple cell structure. It is sensitive to metal toxicity, especially photosynthetic activity during growth [42]. At present, studies on the photosynthetic response of algae cells in response to metal mainly focus on the studies of chlorella, microcystis aeruginosa, and other aquatic environments by Cu^2+^, Zn^2+^, and Mn^2+^ Plasma [43, 44]. This study, to our knowledge, is the first report of exploring genes and metabolic pathways expression profile under cobalt treatment in *B. braunii.* Transcriptomic dynamics tracked via messenger RNA sequencing over four time points during cobalt treatment revealed that under high concentration, the genes that were most strongly expressed were fatty acid biosynthesis and metabolism and oxidative phosphorylation, whereas the most downregulated ones were from carbohydrate metabolism, photosynthesis, and amino acid metabolism. The transcript patterns of global genes showed that diverse expression dynamics probably contributes to the different phenotypes under cobalt enrichment. This study provided us not only a comprehensive picture of metal toxicity adaptive mechanisms from physiological perspective, but also a number of key metabolic nodes might be exploited as target genes for further genetic modification. Thus, these findings serve as foundation for monitoring the metabolic changes in this and related oleaginous algae for an optimized and controllable production of hydrocarbon-based biofuels.

## Materials and methods

### Algae strain and culture conditions

The microalgae species *B. braunii* SAG 807-1 was purchased from SAG culture collection, University of Göttingen, Germany and grown in a modified Chu 13 medium [45]. The alga seed was firstly cultivated with glass bubbling columns (diameter = 5 cm) for about 7 days to prepare the inoculum in tube culture. Each of these columns contain 0.8 L of algal broth and was continuously illuminated by cold-white fluorescent lamps (NFL28-T5, NVC, China) with light intensity of 100 µmol/m^2^/s. The temperature for algal broth was 25 ± 2 °C during the cultivation. Air bubble that contained 1% CO_2_ (v/v) was continuously injected into the bottom of the columns with a speed of 1 vvm to agitate the algal broth as well as supply carbon resource.

### Growth analysis

The biomass concentration of an “algae disk” (DW, g/L) was measured using gravimetric method. Each time 10 mL volume algae cells were washed down and resuspended with deionized water and then filtered to a pre-weighed 0.45 µm GF/C filter membrane (Whatman, England; DW0, mg). The membrane was oven dried at 105 °C for 12 h and then cooled down to room temperature to measure dry weight (DW1, mg). The DW was calculated as follows:

$${\text{DW}} = \, ({\text{DW1 }}{-}{\text{DW}}0)/ 10,$$

where 10 was the volume of each sample.

### Hydrocarbon extraction and analysis

The algal cells were harvested by washing down with de-ionized water and centrifugation at 3800×*g* for 10 min (Allegra X-22R, Beckman coulter, America). The algal pellets were washed three times with de-ionized water to remove the attached salt. Hydrocarbon was determined according to the procedures described in Cheng et al. [12]. Exactly 50 mg of lyophilized cell biomass were homogenized and soaked in *n*-hexane. The extraction process was repeated several times until the supernatant was colorless and then combined in a pre-weighed glass vial. The crude hydrocarbon extract was dried under gentle flow of nitrogen gas (> 99%). The residue remained in the glass vial was considered as the crude hydrocarbon.

### Physiological parameter analysis

The contents of total chlorophyll were determined using a modified method described by Sükran et al. [46]. The centrifuged pellets were resuspended in 96% ethanol and vortexed to extract pigments. Cellular debris was pelleted by centrifugation, and chlorophyll-a and -b levels were determined spectrophotometrically in the supernatant by measuring optical absorbance at 645 and 663 nm.

According to the operating procedures on the Dual-PAM-100 (Walz, Effeltrich, Germany), the maximum photochemical efficiency of photosystem II of algal samples was detected by measuring variable to maximum fluorescence ratio (Fv/Fm). The following operation procedure was according to Fan’s methods [19]. The samples were kept in the dark for 15 min before measurement. The original fluorescence (F0) was determined under the irradiance of measuring light. A saturation pulse was applied to obtain maximum fluorescence (Fm) in the dark-adapted samples. All experiments were conducted in triplicate. The effective PSII quantum yield was calculated as follows:

$${\text{Fv}}/{\text{Fm}}\, = \,\left( {{\text{Fm}} - {\text{F}}0} \right)/{\text{Fm}} .$$

POD activity was determined by the guaiacol methods, and it catalyzed the oxidation of specific substrates by H_2_O_2_ with characteristic light absorption at 470 nm. Glutathione reductase (GR) catalyzed the regeneration of GSH by NADPH reduction GSSG while continuously consuming NADPH to generate NADP^+^. NADPH had a characteristic absorption peak at 340 nm. The rate of NADPH dehydrogenation was determined by measuring the rate of decrease in absorbance at 340 nm and then calculating GR activity. SOD was determined by the xanthine oxidase method (hydroxylamine method). Malondialdehyde (MDA) was determined through visible spectrophotometry. MDA is condensed with TBA to form a red product with a maximum absorption peak at 532, and the absorbance at 600 nm was measured at the same time. The difference in absorbance at 532 nm and 600 nm was used to calculate the content of MDA.

### Electron microscopy observation

To determine the effect of cobalt on cell morphology, cells were fixed and imaged. Cells were collected during the culture time of 0, 4, and 8 days. Sodium phosphate buffer (pH 7.5) to preserve morphologies as well as to prevent any potential chemical degradation. Gently, resuspended cells were imaged with an AxioCam MrC camera (Carl Zeiss, Germany).

### NGS data collection and sequence assembly

Fourteen samples were collected from the bioreactors at 0, 2, 4, and 8 days at both cobalt enrichment and normal conditions and then immediately centrifuged at 6000×*g* for 5 min at 4 °C for later processing. Total RNA was extracted from fresh *B. braunii* cells using RNeasy Mini Kit (Qiagen, Germany) and checked for a RIN (RNA integrity number) to inspect RNA integrity by an Agilent Bioanalyzer 2100 (Agilent technologies, USA). Qualified total RNA was further purified by RNAClean XP Kit (Beckman Coulter, USA) and RNase-Free DNase Set (Qiagen, Germany). The collected RNA solutions were stored at − 80 °C for subsequent library preparation.

Libraries were constructed from about 4 µg of RNA using VAHTS Stranded mRNA-seq Library Prep Kit for Illumina^®^. The peak insert size of each library was about 400–450 bps (Additional file 1: Table S1). The standard Illumina protocol was used in library construction. Subsequently, the mRNA-seq libraries were sequenced on an Illumina HiSeq 2000 platform. Over 6 Giga Raw bases from each library were generated (*Q*20 ratio > 95%) (Additional file 1: Table S2).

Default parameters were used to pass reads using the Seqtk pipeline (https://github.com/lh3/seqtk). After removal of adapters, poly-N strands, ribosome RNA reads, and low-quality reads, all filtered reads (a total of 659 million) were examined by FastQC (http://www.bioinformatics.babraham.ac.uk/projects/fastqc/) to confirm data quality (Additional file 1: Table S3). All the clean reads from both conditions were pooled and subjected to de novo assembly using CLC Genomics Workbench (version: 6.0.4, Word-size = 45, Minimum contig length > 400). CAP3 software [47] were adopted to further generate the final UniGenes (also named ESTs/cDNAs/scaffolds/contigs). As a result, 196,276 non-redundant Unigenes were obtained with average length of 1086 bp, N50 of 1419 bp, and GC content of 51.76% (Table 1).

The mRNA-seq paired-end sequencing data are available at the NCBI’s Sequence Read Archive database with an accession number SRP161189.

### Transcriptome functional annotation and differential expression analysis

The general unigenes (unique transcripts) were subjected to sequence homology comparison against the NCBI non-redundant protein database and UniProt using the BlastX algorithm with a cut-off *E*-value ≤ 10^−5^. Gene Ontology (GO) classification of each gene model was carried out using Blast2go software. KOG (eukaryotic ortholog group) and COG (cluster of orthologous groups of proteins) analysis were carried out using rpstblastn program. Kyoto encyclopedia of genes and genomes (KEGG) classification was performed using KASS and KEGG automatic annotation sever.

Reads counts per unigene and its normalized level of FPKM (fragments per kilobase of exon model per million mapped reads) were obtained using eXpress software. Differential expression analysis of the samples was conducted using the edgeR software [48], with a threshold *q*-value of ≤ 0.005. Fold changes between different time points were calculated using log_2_ ratios. Genes were regarded as differentially expressed when these showed at least a twofold change and ≤ 5% false discovery rate (*q* value). GO and KEGG pathway enrichment were analyzed based on hypergeometric test and Bonferroni adjustment (corrected *P* value (FDR) ≤ 0.05).

### Quantitative real-time PCR validation

To verify the results of mRNA-seq analysis, ten unigenes were randomly selected for validation by using standard qRT-PCR 2^−ΔΔCT^ methods. First-strand cDNA synthesis and qRT-PCR were performed using the ReverTra Ace qPCR Kit (TOYOBO, FSQ-101) with gDNA Remover and Power SYBR Green PCR Master Mix (ABI, 4368708), respectively. The EF-1 alpha gene was used as internal control for normalization. All PCR reactions were performed on a QuantStudio 5 Real-Time PCR System (ABI, USA). The primers pairs used for qPCR analyses and comparison results with FPKM values were listed in Additional file 14: Dataset S13.

### Statistical analysis

All the algal growth experiments were repeated three times independently, and data were measured as the mean with standard deviation (SD). For mRNA-sequencing, two separate biological replicates were analyzed in parallel. Statistical analyses were performed using the Spearman correlation analysis (SPSS19.0). For all of the data analysis, *P* value < 0.05 was considered statistically significant.

## Additional files

**Additional file 1.** Additional Tables S1–S4 and Figures S1–S12.

**Additional file 2: Dataset S1.** Annotation of the de novo-assembled unigenes from NCBI nr database.

**Additional file 3: Dataset S2.** Annotation of the de novo-assembled unigenes from Gene Ontology database.

**Additional file 4: Dataset S3.** KEGG pathway annotation of the de novo-assembled unigenes.

**Additional file 5: Dataset S4.** Unigene counts of GO enrichment upon high cobalt treatment (2 days).

**Additional file 6: Dataset S5.** Unigene counts of GO enrichment upon high cobalt treatment (4 days).

**Additional file 7: Dataset S6.** Unigene counts of GO enrichment upon high cobalt treatment (8 days).

**Additional file 8: Dataset S7.** KEGG metabolic pathways enrichment upon high cobalt treatment (2 days).

**Additional file 9: Dataset S8.** KEGG metabolic pathways enrichment upon high cobalt treatment (4 days).

**Additional file 10: Dataset S9.** KEGG metabolic pathways enrichment upon high cobalt treatment (8 days).

**Additional file 11: Dataset S10.** List of unigenes that differentially expressed upon high cobalt treatment (2 days).

**Additional file 12: Dataset S11.** List of unigenes that differentially expressed upon high cobalt treatment (4 days).

**Additional file 13: Dataset S12.** List of unigenes that differentially expressed upon high cobalt treatment (8 days).

**Additional file 14: Dataset S13.** Comparison of qRT-PCR with RNA-seq results using 10 randomly selected unigenes.

## Abbreviations

NGS: next generation sequencing
KOG: eukaryotic ortholog group
COG: cluster of orthologous groups of proteins
GO: gene ontology
KEGG: kyoto encyclopedia of genes and genomes
TAG: triacylglycerols
VLCFA: very long chain fatty acids
POD: peroxidase
GR: glutathione reductase
SOD: superoxide dismutase
MDA: malonaldehyde
FPKM: fragments per kilobase of exon model per million mapped reads
PDAT: phospholipid:diacylglycerol acyltransferase
DW: dry weight
