# Supplementary material for: Comparative transcriptome analyses of oleaginous Botryococcus braunii race A reveal significant differences in gene expression upon cobalt enrichment
Source: Biotechnol Biofuels. 2018 Dec 18;11:333. doi: 10.1186/s13068-018-1331-5 (PMC6297975; doi:10.1186/s13068-018-1331-5)
Supplement: Supplementary file 1 — Additional file 1. Additional Tables S1–S4 and Figures S1–S12. [file 13068_2018_1331_MOESM1_ESM.docx]

**Table S1 Qubit® 2.0 Fluorometer quantitative results of *B. braunii* SAG 807-1.**

| **No.** | **Sample Name** | **Index No.** | **Con. (ng/μL)** | **Peak Lenth. (bp)** |
| --- | --- | --- | --- | --- |
| 1 | 0-0d-1 | V81 | 6.9 | 445 |
| 2 | 0-0d-2 | V82 | 54.8 | 433 |
| 3 | G-2d-1 | V83 | 30.2 | 440 |
| 4 | G-2d-2 | V84 | 22 | 432 |
| 5 | Z-2d-1 | V85 | 2.22 | 441 |
| 6 | Z-2d-2 | V86 | 25.4 | 442 |
| 7 | G-4d-1 | V87 | 21.4 | 447 |
| 8 | G-4d-2 | V88 | 14.4 | 443 |
| 9 | Z-4d-1 | V89 | 12.4 | 447 |
| 10 | Z-4d-2 | V90 | 10.2 | 428 |
| 11 | G-8d-1 | V91 | 19.7 | 439 |
| 12 | G-8d-2 | V92 | 22 | 442 |
| 13 | Z-8d-1 | V93 | 32 | 431 |
| 14 | Z-8d-2 | V94 | 32.6 | 427 |

**Table S2 Quality control results of mRNA-seq from *B. braunii* SAG 807-1.**

| **Sample Name** | **Seq. type** | **Orientation** | **Raw reads (M)** | **Raw bases(G)** | **Q20 ratio (%)** |
| --- | --- | --- | --- | --- | --- |
| G-2d-1 | mRNA | Forward/Reverse | 45.20 | 6.78 | 96.50 |
| G-2d-2 | mRNA | Forward/Reverse | 45.00 | 6.75 | 96.69 |
| G-4d-1 | mRNA | Forward/Reverse | 48.93 | 7.34 | 96.55 |
| G-4d-2 | mRNA | Forward/Reverse | 53.53 | 8.03 | 96.78 |
| G-8d-1 | mRNA | Forward/Reverse | 46.80 | 7.02 | 96.72 |
| G-8d-2 | mRNA | Forward/Reverse | 49.60 | 7.44 | 96.44 |
| Z-2d-1 | mRNA | Forward/Reverse | 61.60 | 9.24 | 96.29 |
| Z-2d-2 | mRNA | Forward/Reverse | 49.60 | 7.44 | 96.66 |
| Z-4d-1 | mRNA | Forward/Reverse | 49.87 | 7.48 | 96.51 |
| Z-4d-2 | mRNA | Forward/Reverse | 46.60 | 6.99 | 96.70 |
| Z-8d-1 | mRNA | Forward/Reverse | 52.33 | 7.85 | 96.66 |
| Z-8d-2 | mRNA | Forward/Reverse | 52.00 | 7.80 | 96.70 |
| 0-0d-1 | mRNA | Forward/Reverse | 45.47 | 6.82 | 96.31 |
| 0-0d-2 | mRNA | Forward/Reverse | 41.53 | 6.23 | 96.64 |

Note：Q20=bases of Q>=20 / all bases of sequencing

**Table S3 The result of sequencing data pre-processing of *B. braunii* SAG 807-1.**

| **Sample ID** | **Raw reads** | **Clean reads** | **Clean ratio** | **rRNA trimed** | **rRNA ratio** |
| --- | --- | --- | --- | --- | --- |
| 0-0d-1 | 45451144 | 43306050 | 95.28% | 43202325 | 0.24% |
| 0-0d-2 | 41508952 | 40247485 | 96.96% | 40220358 | 0.07% |
| G-2d-1 | 45199444 | 43385877 | 95.99% | 43253546 | 0.31% |
| G-2d-2 | 44986818 | 43206753 | 96.04% | 43019901 | 0.43% |
| G-4d-1 | 48915652 | 46935507 | 95.95% | 46816345 | 0.25% |
| G-4d-2 | 53534456 | 51602856 | 96.39% | 51513699 | 0.17% |
| G-8d-1 | 46820052 | 45012440 | 96.14% | 44941758 | 0.16% |
| G-8d-2 | 49608076 | 47633470 | 96.02% | 47560767 | 0.15% |
| Z-2d-1 | 61592150 | 57572917 | 93.47% | 57340427 | 0.40% |
| Z-2d-2 | 49584358 | 47576477 | 95.95% | 47342425 | 0.49% |
| Z-4d-1 | 49889212 | 47768939 | 95.75% | 47622748 | 0.31% |
| Z-4d-2 | 46579854 | 44587238 | 95.72% | 44447466 | 0.31% |
| Z-8d-1 | 52355938 | 50402094 | 96.27% | 50341757 | 0.12% |
| Z-8d-2 | 52021972 | 50062885 | 96.23% | 50003050 | 0.12% |

Clean ratio=(Clean reads/Raw reads)%; rRNA ratio=[(Clean reads - rRNA trimed)/ Clean reads]%

**Table S4 Unigenes annotation results of *B. braunii* SAG 807-1.**

| **Database** | **Total Unigenes** | **Annotated** | **Ratio** |
| --- | --- | --- | --- |
| NR | 196276 | 76002 | 38.72% |
| Uniprot | 196276 | 84847 | 43.23% |


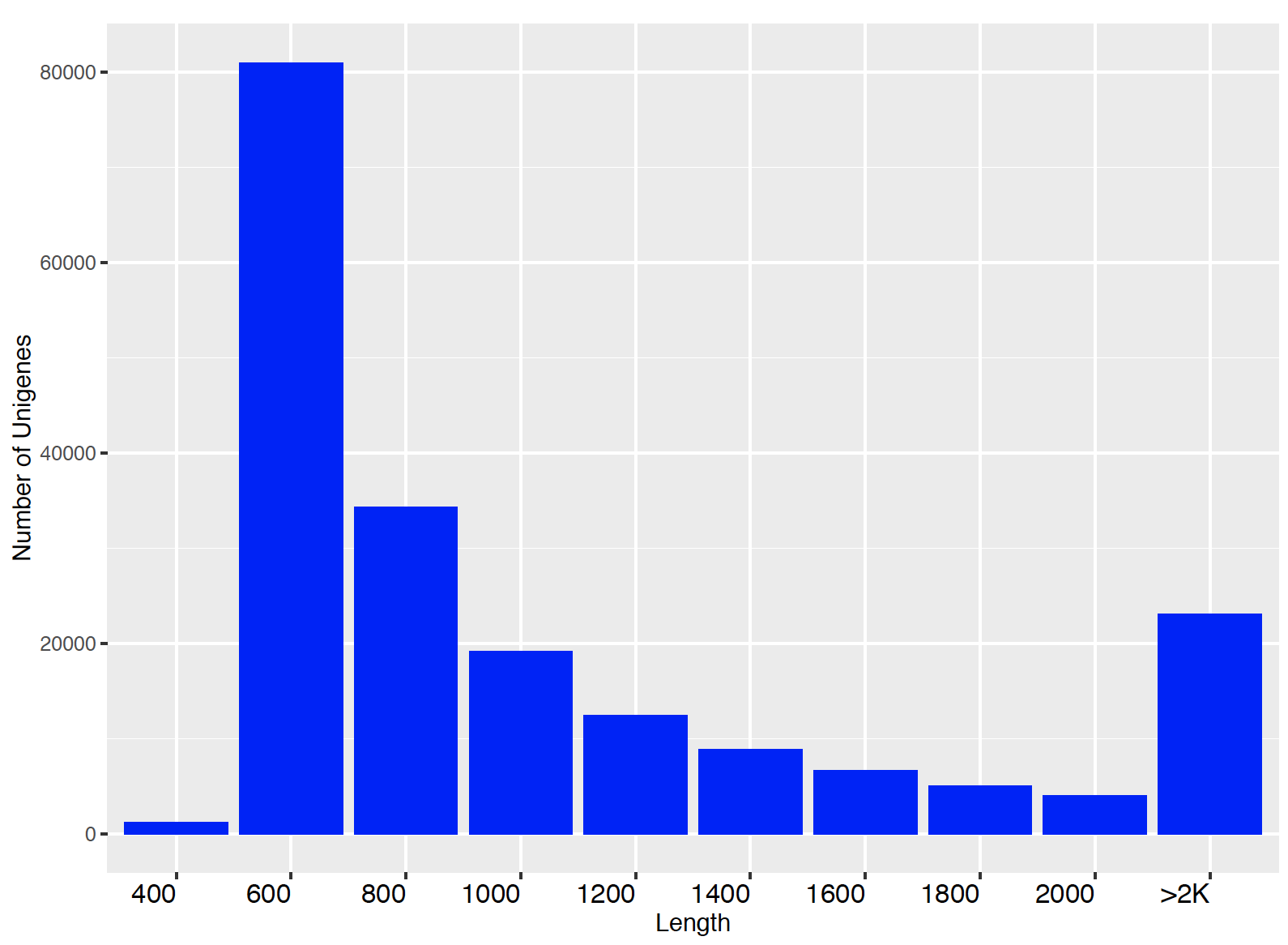


**Figure S1 Final unigenes length distribution of *B. braunii* SAG 807-1.**


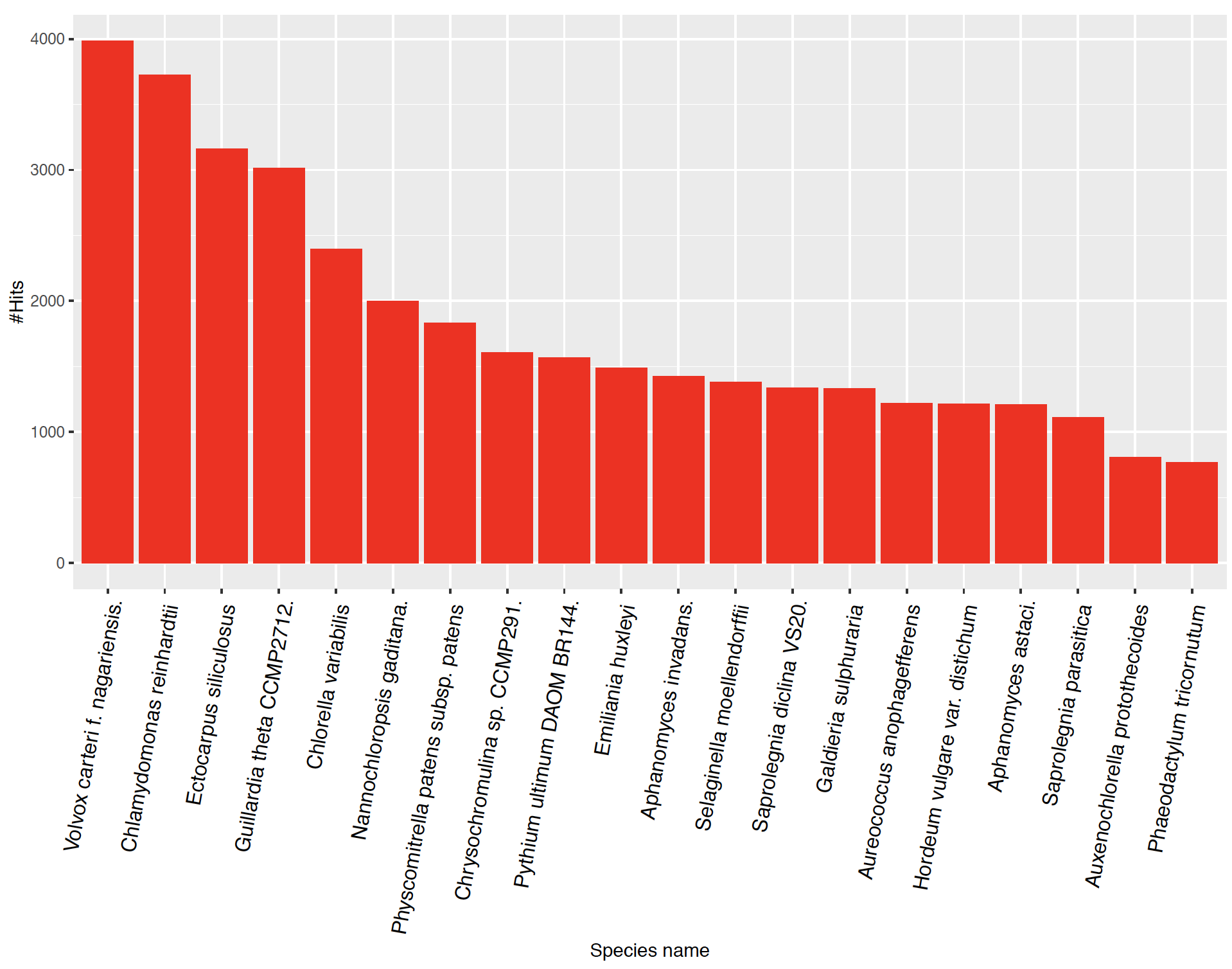


**Figure S2 Distribution of number of best-hits derived from the genome of top 20 species.**


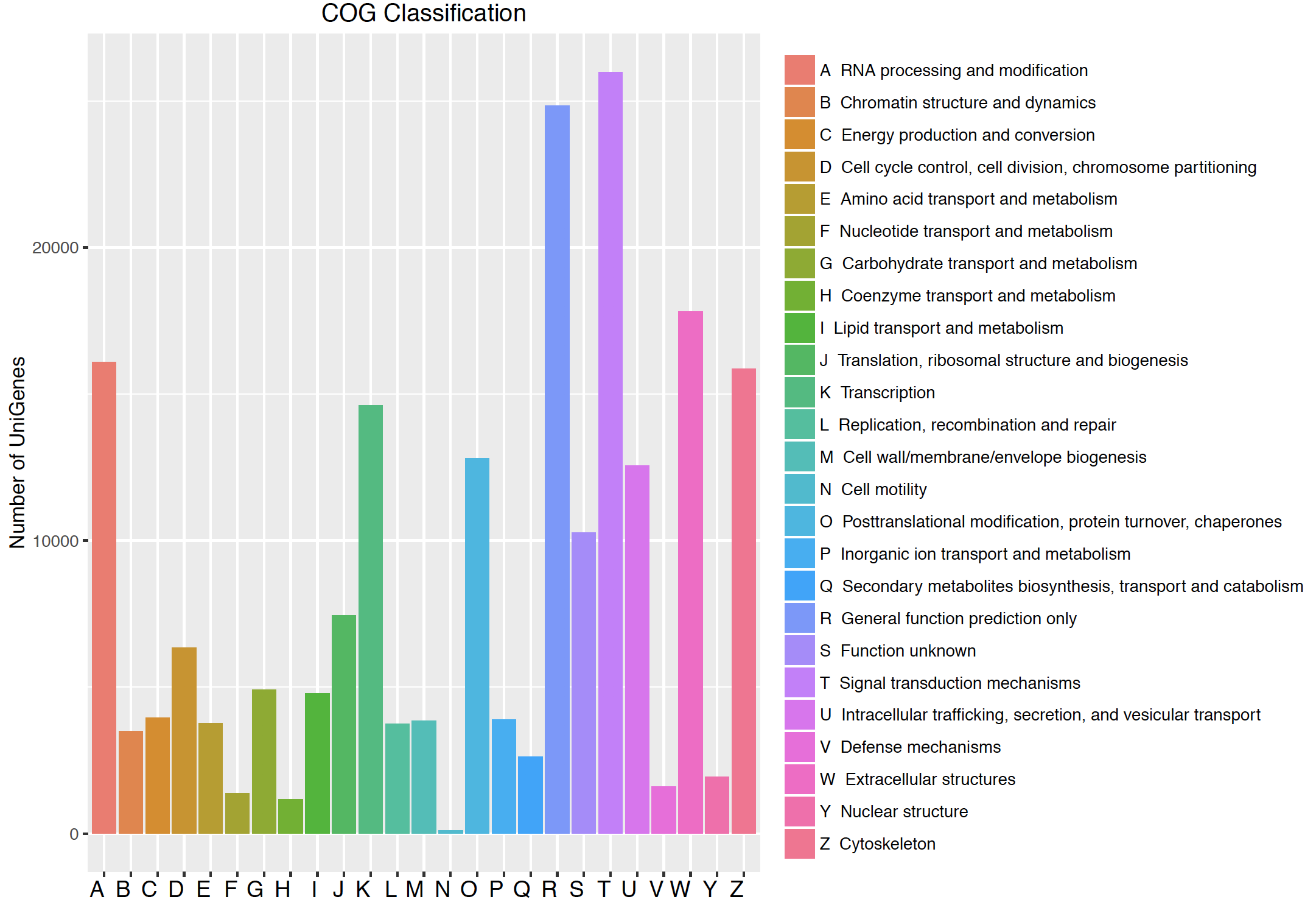


**Figure S3 COG classification of the de novo-assembled unigenes from *B. braunii* SAG 807-1.**


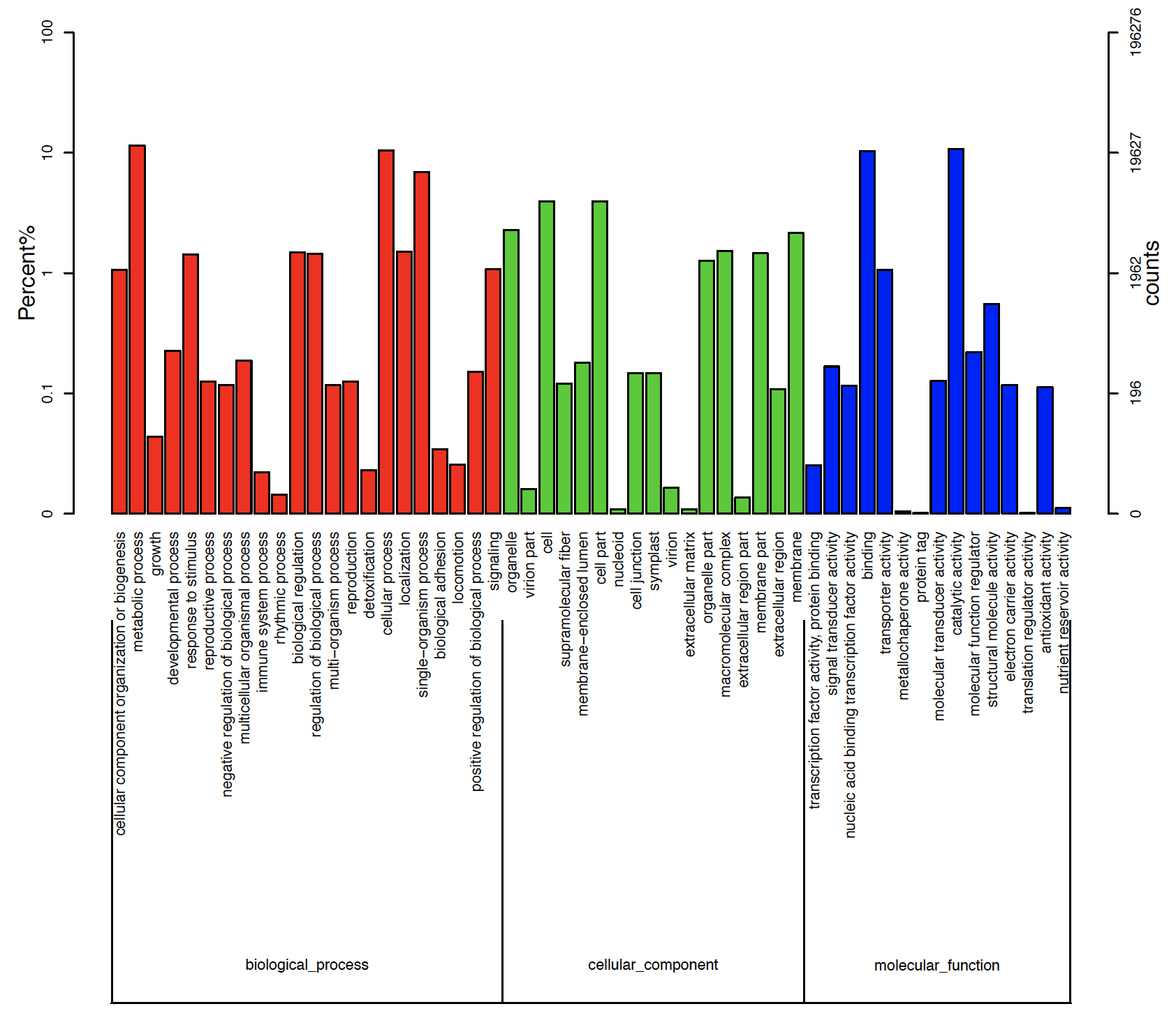


**Figure S4 Gene Ontology (GO) annotation of unigenes obtained from the *B. braunii* SAG 807-1 transcriptome.**


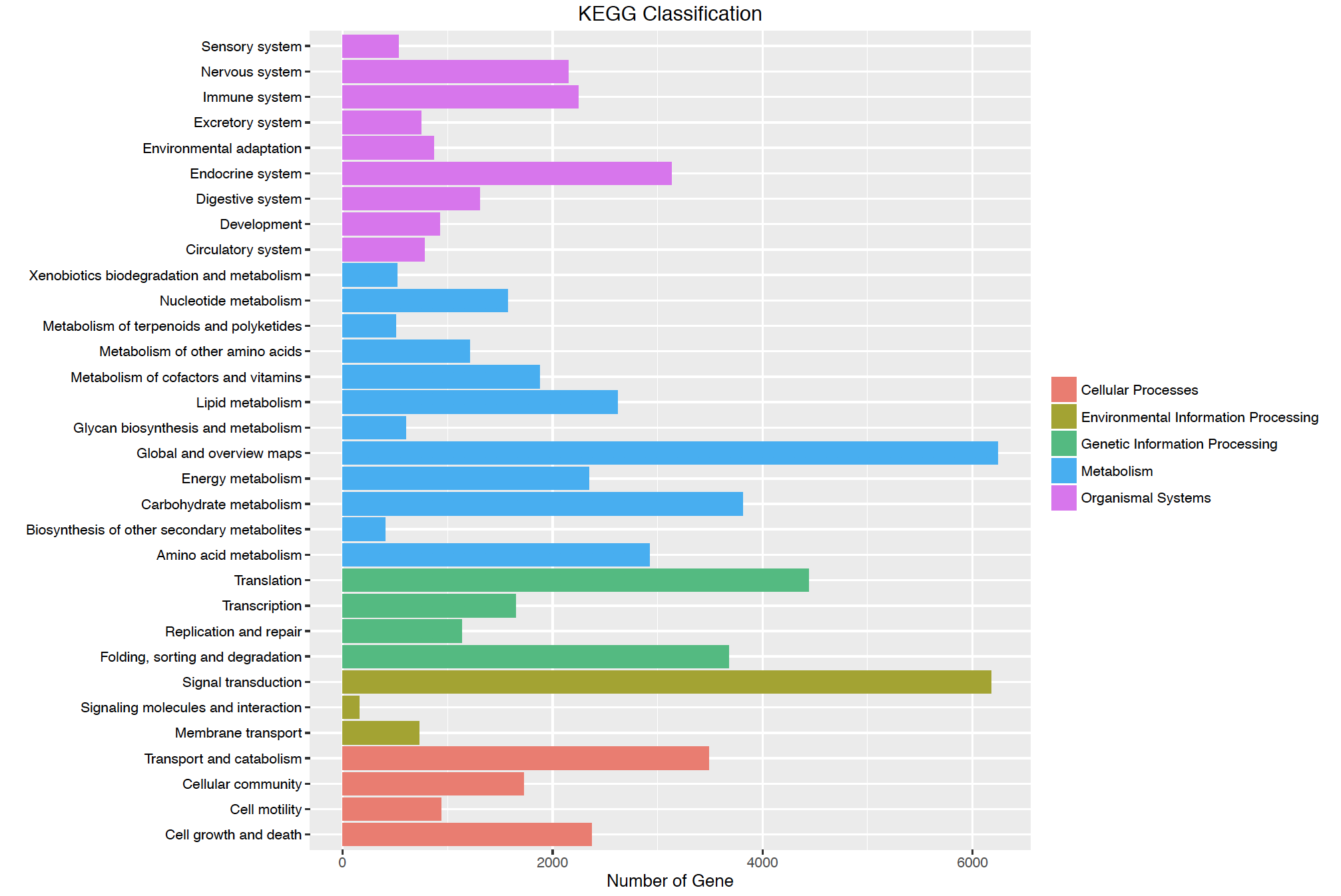


**Figure S5 KEGG classification of the de novo-assembled unigenes from *B. braunii* SAG 807-1.**

**Figure S6 Number of differentially expressed genes of three time points upon cobalt enrichment treatment.**

**
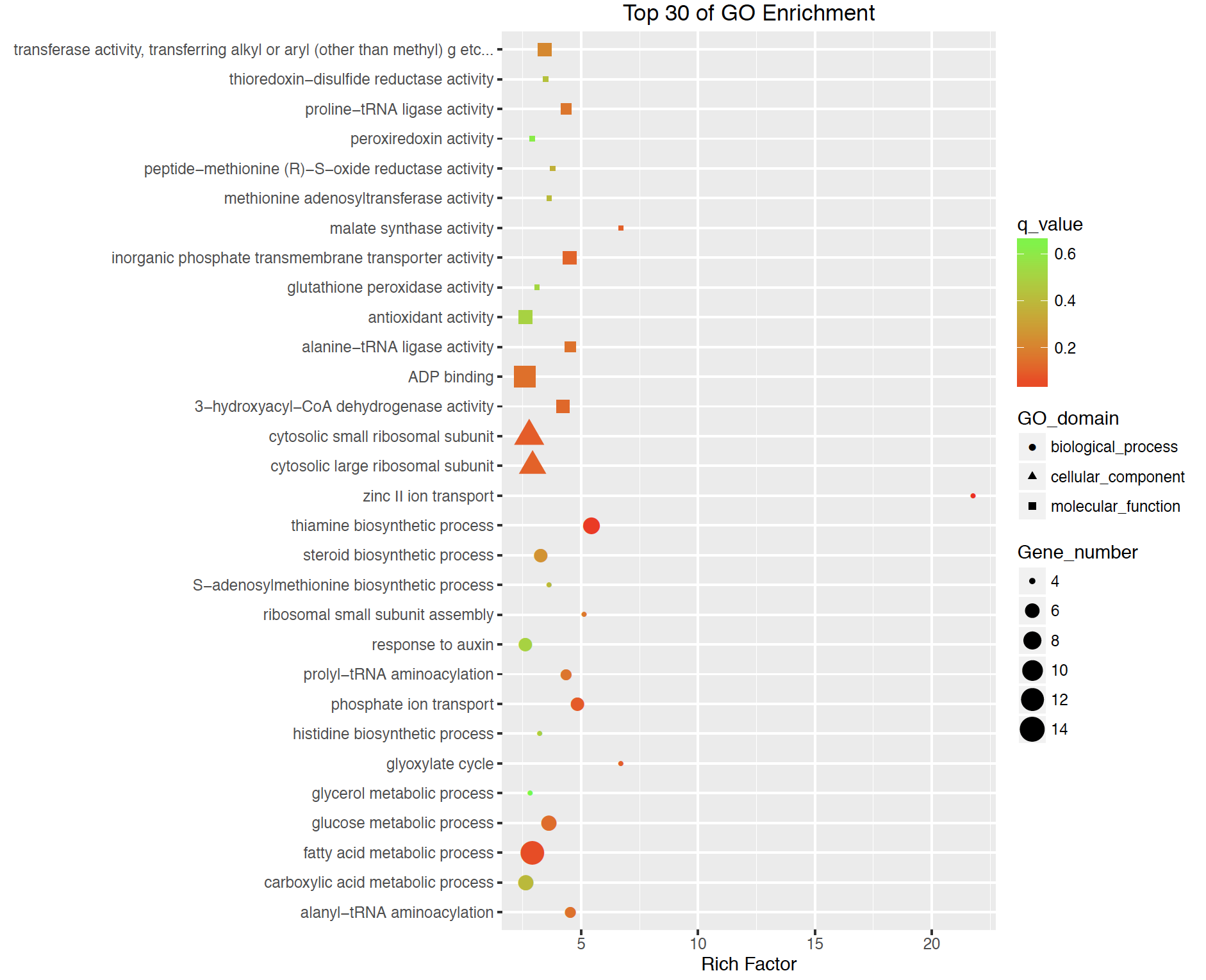
**

**Figure S7 Distribution of top 30 GO categories enrichment upon high cobalt treatment (2 days).** Enrichment were analyzed based on hypergeometric test and Bonferroni adjustment (corrected *p*-value (FDR) ≤0.05).

**
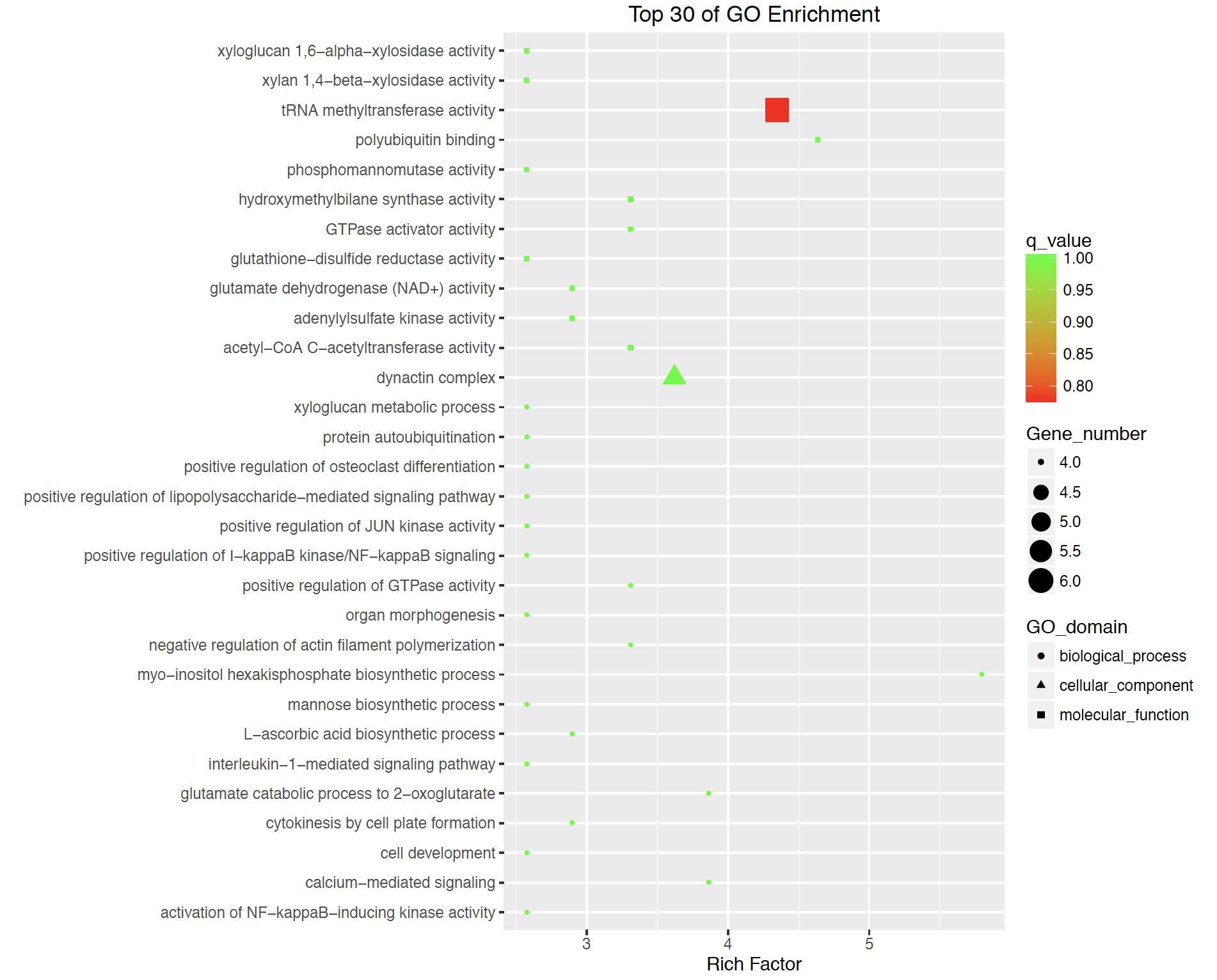
**

**Figure S8 Distribution of top 30 GO categories enrichment upon high cobalt treatment (8 days).** Enrichment were analyzed based on hypergeometric test and Bonferroni adjustment (corrected *p*-value (FDR) ≤0.05).

**
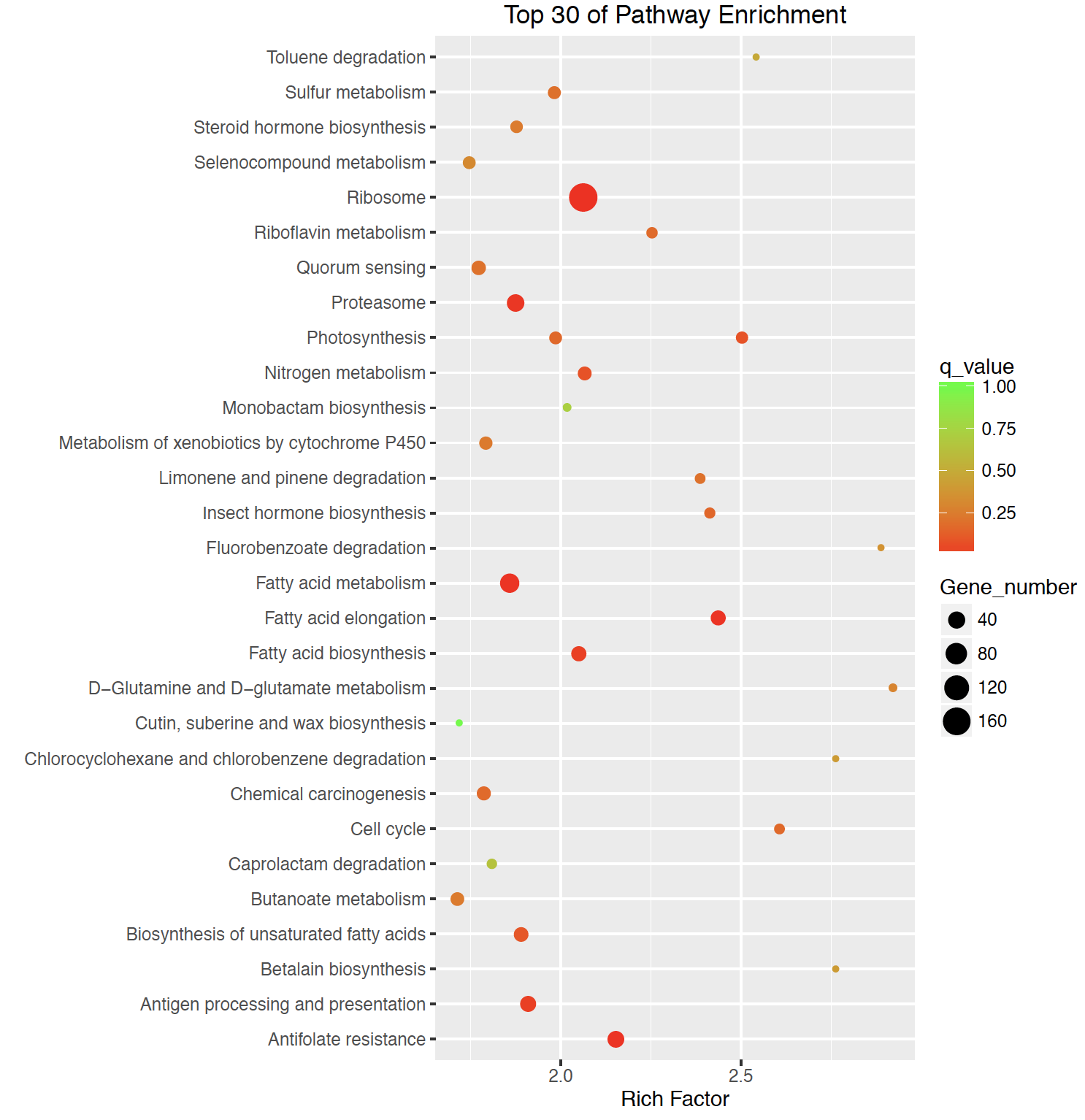
**

**Figure S9 Distribution of top 30 KEGG metabolic pathways enrichment upon high cobalt treatment (2 days).** Enrichment were analyzed based on hypergeometric test and Bonferroni adjustment (corrected *p*-value (FDR) ≤0.05).

**
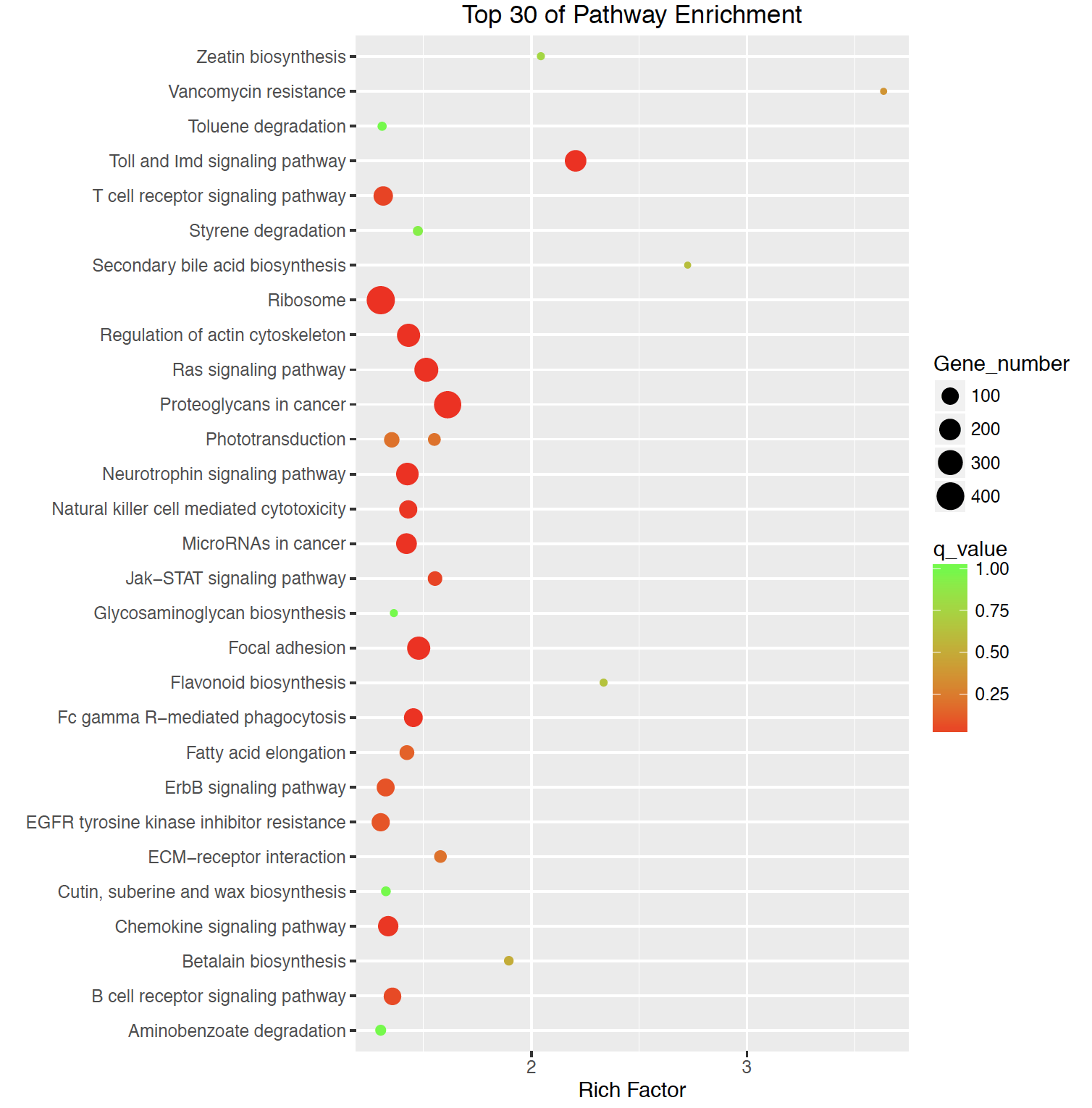
**

**Figure S10 Distribution of top 30 KEGG metabolic pathways enrichment upon high cobalt treatment (8 days).** Enrichment were analyzed based on hypergeometric test and Bonferroni adjustment (corrected *p*-value (FDR) ≤0.05).


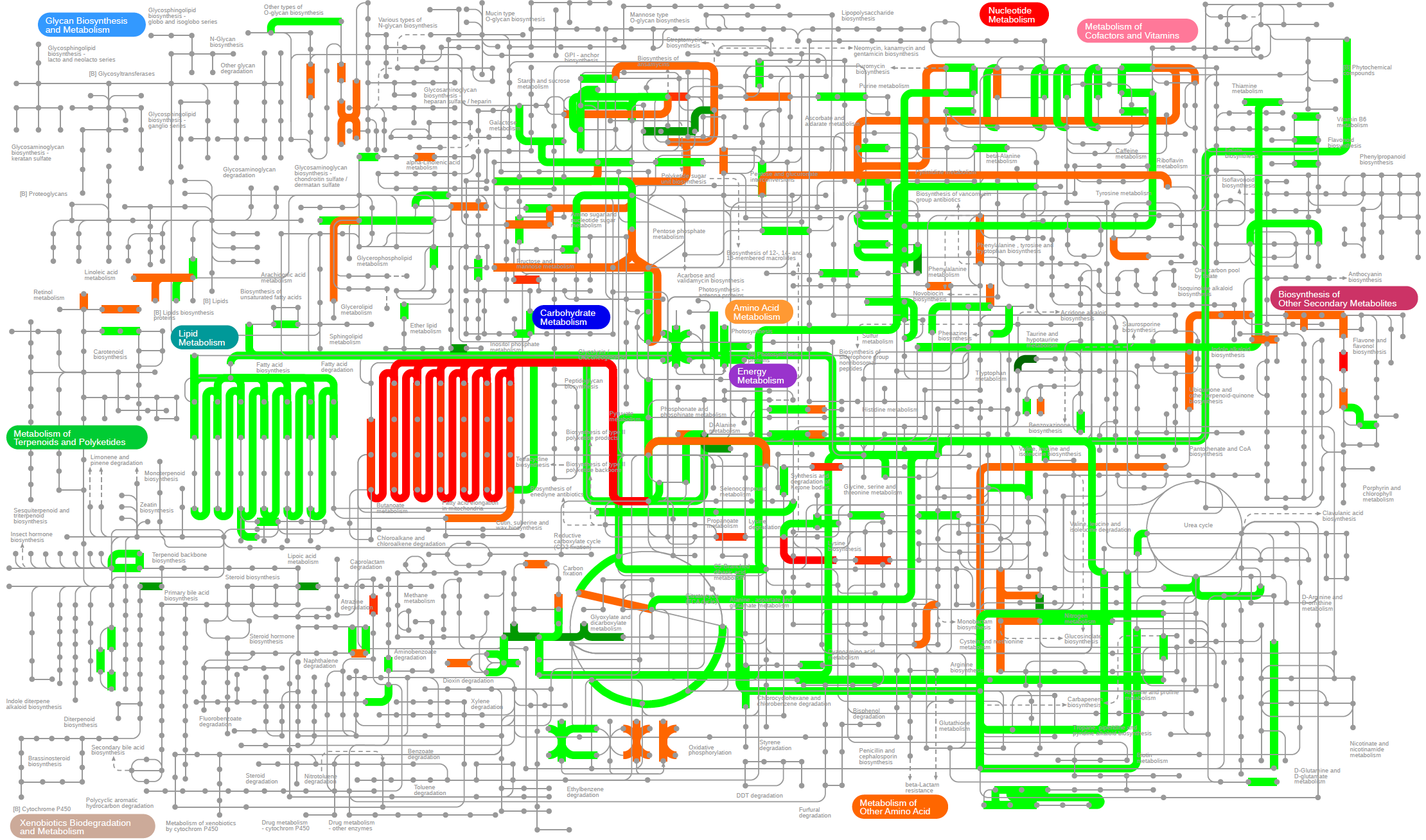


**Figure S11 Overview of metabolic pathways and regulation during the high cobalt treatment in *B. braunii* SAG 807-1 (4 days).** Pathways that are up- or down-regulated (compared with normal culture conditions) are labeled in red and green, respectively. Deeper red and green indicates greater fold changes of differential expression. These map were visualized by iPath 3 interactive pathways explorer.


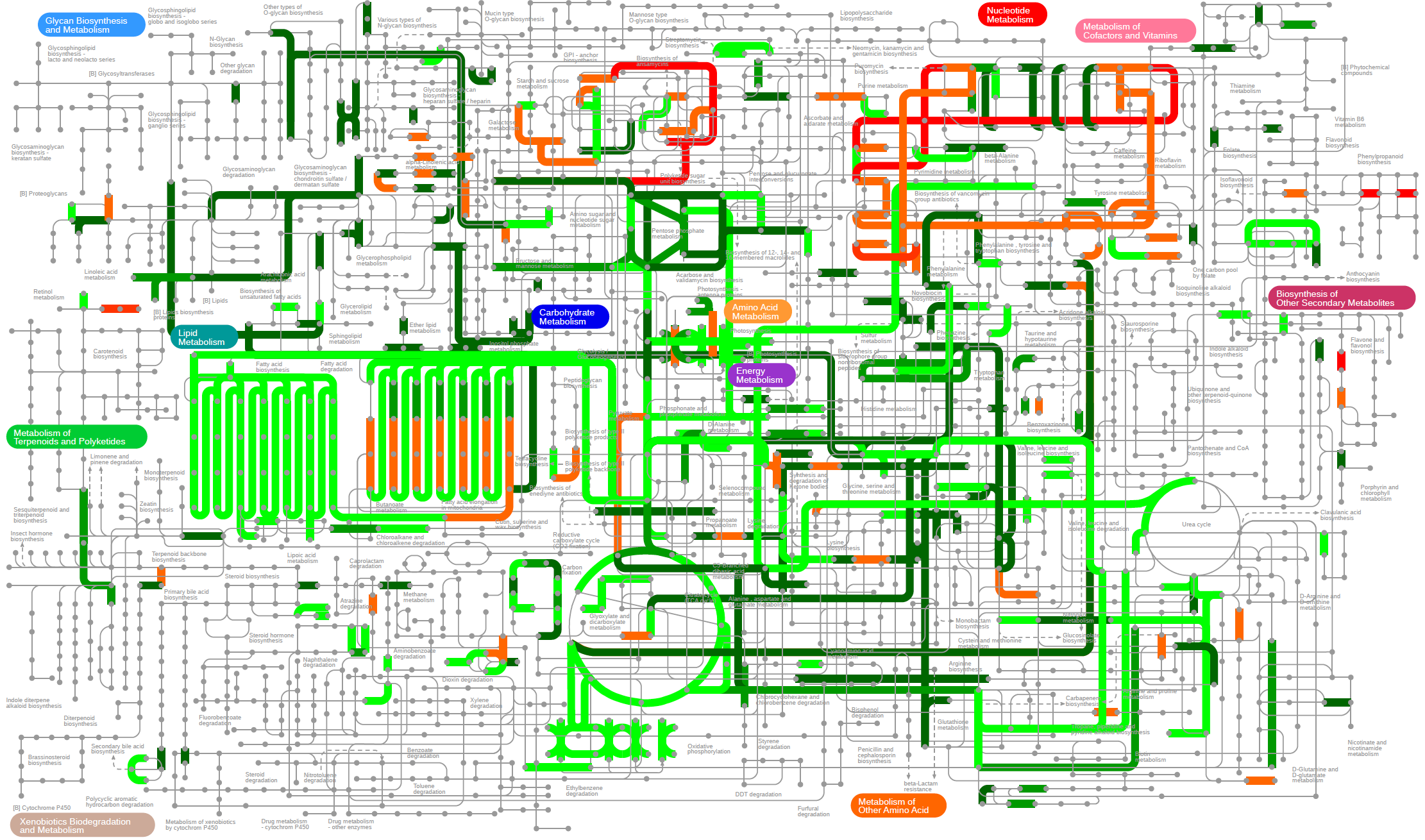


**Figure S12 Overview of metabolic pathways and regulation during the high cobalt treatment in *B. braunii* SAG 807-1 (8 days).** Pathways that are up- or down-regulated (compared with normal culture conditions) are labeled in red and green, respectively. Deeper red and green indicates greater fold changes of differential expression. These map were visualized by iPath 3 interactive pathways explorer.
